# Pathogenic Lifestyles of *E. coli* Pathotypes in a Standardized Epithelial Cell Model Influence Inflammatory Signaling Pathways and Cytokines Secretion

**DOI:** 10.3389/fcimb.2016.00120

**Published:** 2016-10-07

**Authors:** Javier Sanchez-Villamil, Gabriela Tapia-Pastrana, Fernando Navarro-Garcia

**Affiliations:** Department of Cell Biology, Centro de Investigación y de Estudios Avanzados del Instituto Politécnico NacionalMéxico City, Mexico

**Keywords:** NF-κB, ERK1/2, pathogenic *E. coli*, inflammatory response, IL-8, TNF-α

## Abstract

Inflammatory response is key for the host defense against diarrheagenic *Escherichia coli* and contributes to the pathogenesis of the disease but there is not a comparative study among different diarrheagenic pathotypes. We analyzed the inflammatory response induced by five diarrheagenic pathotypes in a HT-29 cell infection model. The model was unified to reproduce the pathogenesis of each pathotype. To compare the inflammatory responses we evaluated: (i) nuclear NF-κB and ERK1/2 translocation by confocal microscopy; (ii) kinetics of activation by each pathway detecting p65 and ERK1/2 phosphorylation by Western blotting; (iii) pathways modulation through bacterial infections with or without co-stimulation with TNF-α or EGF; (iv) cytokine profile induced by each pathotype with and without inhibitors of each pathway. EHEC but mainly EPEC inhibited translocation and activation of p65 and ERK1/2 pathways, as well as cytokines secretion; inhibition of p65 and ERK1/2 phosphorylation prevailed in the presence of TNF-α and EGF, respectively. Intracellular strains, EIEC/*Shigella flexneri*, caused a strong translocation, activation, and cytokines secretion but they could not inhibit TNF-α and EGF stimulation. ETEC and mainly EAEC caused a moderate translocation, but a differential activation, and high cytokines secretion; interestingly TNF-α and EGF stimulation did no modify p65 and ERK1/2 activation. The use of inhibitors of NF-κB and/or ERK1/2 showed that NF-κB is crucial for cytokine induction by the different pathotypes; only partially depended on ERK1/2 activation. Thus, in spite of their differences, the pathotypes can also be divided in three groups according to their inflammatory response as those (i) that inject effectors to cause A/E lesion, which are able to inhibit NF-κB and ERK1/2 pathways, and cytokine secretion; (ii) with fimbrial adherence and toxin secretion with a moderate inhibition of both pathways but high cytokines secretion through autocrine cytokine regulation; and (iii) the intracellular bacteria that induce the highest pathways activation and cytokines secretion by using different activation mechanisms. This study provides a comprehensive analysis of how the different pathogenesis schemes of *E. coli* pathotypes manipulate inflammatory signaling pathways, which leads to a specific proinflammatory cytokine secretion in a cell model infection that reproduce the hallmarks of infection of each pathotype.

## Introduction

Diarrheal diseases are responsible of approximately 800,000 deaths per year in children under 5 years old and represent 10% of deaths worldwide (Liu et al., 2012) and the second cause of death (Lanata et al., 2013). Rotavirus, calicivirus, enteropathogenic, and enterotoxigenic *Escherichia coli* cause more than a half of all deaths by diarrhea in children under 5 years old (Lanata et al., 2013). There are six main pathotypes of diarrheagenic *E. coli*: enteropathogenic *E. coli* (EPEC), enterohemorrhagic *E. coli* (EHEC), enterotoxigenic *E. coli* (ETEC), enteroaggregative *E. coli* (EAEC), diffusely adherent *E. coli* (DAEC), and enteroinvasive *E. coli* (EIEC). The clinical symptoms of each pathotype differ, as well as colonization site, infection mechanism, and thereby the induced diseases are different (Croxen et al., 2013), this exemplifies the *E. coli* diversity, which includes intra and extracellular pathotypes. Diarrheagenic *E. coli* pathotypes secrete diverse toxins, effectors and virulence factors for exploiting host cell functions for their colonization. *E. coli* pathotypes can be grouped by some similarity in their pathogenic mechanisms. EPEC and EHEC are grouped as pathogens that induced an intestinal lesion, named attaching and effacing lesion (A/E lesion). A/E pathogens are intimately adhered to intestinal epithelial cells (IECs), causing localized elimination of microvilli and accumulation of cytoskeletal proteins underneath adhered bacteria, called pedestals (McDaniel et al., 1995). EHEC is distinguished from EPEC by the presence of the Shiga toxin (Stx), which is cytotoxic and responsible for the fatal hemolytic uremic syndrome (Croxen et al., 2013). ETEC and EAEC are a common cause of travelers' diarrhea; ETEC is defined for elaborating the heat-labile enterotoxin (LT) and/or the heat-stable enterotoxin (ST; Huang et al., 2004), and EAEC has been defined by its phenotype of aggregative adherence to HEp-2 cells (Nataro et al., 1995). EAEC produces enterotoxic and cytotoxic effects such as intestinal crypts dilatation, enterocytes rounding, and extrusion (Estrada-Garcia and Navarro-Garcia, 2012). EIEC is phylogenetically closely related to *Shigella* spp. and have a virulence plasmid (pINV), which is essential for the invasive phenotype (Croxen et al., 2013). However, the infection induced by EIEC is lesser severe than that induced by *Shigella* (DuPont et al., 1989), which has been associated to a low expression of virulence factors by EIEC on the host cell (Moreno et al., 2009).

Diarrheagenic *E. coli* provide an interesting model to study the inflammatory response induced by enteropathogens, since *E. coli* strains have acquired diverse mobile genetic elements due to their genome plasticity, which allows having different pathotypes in the same bacterial species. Besides, all pathotypes have diverse pathogen-associated molecular patterns (PAMPs) that are recognized by pattern recognition receptors (PRRs). IECs work as sensors detecting PAMPs, through PRRs, as extracellular and intracellular receptors: Toll-like receptors (TLRs) and NOD-like receptors (NLRs; Kagnoff and Eckmann, 1997). PRRs stimulation activates signaling cascades of nuclear factor κB (NF-κB) and mitogen activated protein kinases (MAPK), which are fundamental for an effective immune response. NF-κB p65/p50 complex is known as the classical o canonical pathway that regulates gene expression involved in the inflammatory response (Gasparini and Feldmann, 2012). NF-κB is in inactive form in the cytoplasm by binding to the inhibitory protein, IκB. Stimulation by various inductors activates a signaling cascade that culminates in IκB phosphorylation resulting in IκB degradation. NF-κB is released and translocated into the nucleus, where it activates various genes that together regulate the inflammatory response (Kawai and Akira, 2010). Activation of NF-κB is dependent on MAPKs that are central in various cellular responses including cytokines regulation. There are three main groups of MAPKs: ERK1/2, JNK, and p38. ERK1/2 are activated by MAP kinase kinase (MKK) and MKK2, JNK by MKK4 and MKK7, and p38 by MKK3, MKK4, and MKK6. After activation of MAPKs, transcription factors in the cytoplasm or nucleus are phosphorylated and activated, leading to the gene expression as a cellular response (Arthur and Ley, 2013).

Inflammatory response studies by each diarrheagenic pathotype have been performed in different epithelial cell lines and infection conditions, which have led to a particular inflammatory response and sometimes the cell models are inappropriate for comparing the diverse inflammatory responses; in term of protein expression, receptors, inflammatory mediators among other characteristics (Sanchez-Villamil and Navarro-Garcia, 2015). For instance, Elewaut et al. showed differences in the degradation activity on IκBα and IκBε among cell lines (Caco-2, HT-29, or T84 cell) infected with EIEC, as well as different inflammatory responses in these cells stimulated by TNF-α (Elewaut et al., 1999). Additionally, there are not studies for comparing the inflammatory responses induced by the different *E. coli* pathotypes under similar conditions, even though many works in an individual way claim a strong induction of the inflammatory response by these pathotypes. Thus, a fundamental question is how the life-style of each pathotype influences the inflammatory response independently to belong of the same species, which can lead to a particular intestinal cytokine microenvironmental. In this work, we developed an intestinal epithelial cell model to study pro- and anti-inflammatory responses induced by the different diarrheagenic *E. coli* pathotypes. This standardized cell model allowed us to compare the different inflammatory responses induced in epithelial cells showing the phenotypic hallmark by the infection with each pathotype. The inflammatory response induced by each pathotype was different regarding to nuclear translocation of phosphorylated p65 (NF-κB) and p44 and p42 (ERK1/2) as well as their kinetics of phosphorylation, modulation of the stimulus with TNF-α (NF-κB), and EGF (ERK1/2) and cytokines secretion in the presence or absence of inhibitors for NF-κB and ERK1/2.

## Materials and methods

### Bacterial strains

Bacterial strains used in this study are described in Table 1. Extracellular bacterial strains were preserved at −70°C in LB with 10% glycerol and intracellular bacteria were grown in TSB (tryptic soy broth). For each experiment, bacteria were inoculated in LB or TSB and incubated overnight at 37°C. Before cell infection, the overnight cultures were activated in DMEM medium without fetal bovine serum (FBS) and without antibiotics and incubated for 2 h at 37°C in static.

**Table 1:** **Bacterial strains used in this study**.

***E. coli* pathotype**	**Strain**	**Description**	**Adhesion**	**References**
EPEC	E2348/69	Prototypical EPEC O127:H6	Attaching and effacing	Levine et al., 1978
EHEC	EDL933	Prototypical EHEC O157:H7	Attaching and effacing	Riley et al., 1983
ETEC	H104O7	Prototypical ETEC O78:H11	CF mediated	Evans et al., 1975
EAEC	O42	Prototypical EAEC O44:H18	Stacked brick	Nataro et al., 1995
EIEC	E11	Prototypical EIEC O124:NM	Invasive	DuPont et al., 1971
***Shigella*** **spp**.				
*S. flexneri*	2457T	Serotype 2a	Invasive	Labrec et al., 1964
**Non-pathogenic**				
*E. coli* K12	HB101	Non-pathogenic K12 strain	NA	Boyer and Roulland-Dussoix, 1969

*NA, not applicable*.

### Cell lines

The HT-29 human colorectal adenocarcinoma cell line (ATCC HTB-38) was grown in DMEM (Gibco, life technologies, Carlsbad, CA, USA) supplemented with 0.1 mM non-essential amino acids, 100 U of penicillin/ml, 100 μg of streptomycin/ml, and 10% FBS (Biowest, Nuaillé, France). The THP-1 human acute monocytic leukemia cell line (ATCC TIB-202) was cultured in RPMI 1640 medium with 100 U of penicillin/ml and 100 μg of streptomycin/ml and 10% FBS. Both cell lines were incubated at 37°C in a 5% CO_2_ incubator. THP-1 cells were differentiated into macrophage-like cells with 10 ng/ml of phorbol 12-myristate 13-acetate (PMA; Sigma-Aldrich) during 48 h.

### Infection model

#### For analysis by confocal microscopy

HT-29 cells were cultured in Labtek slides (VWR, Batavia, IL, USA) and incubated for 48 h to reach a 90% of confluence. Cells were washed and then were starved by adding DMEM without FBS and antibiotics and then incubated for another 24 h as a standard method to avoid undesired stimulation by serum components. Before interaction, cells were washed and kept in DMEM without FBS and antibiotics. Cells were inoculated with the corresponding bacterial cultures at a multiplicity of infection (MOI) of 10 and incubated for 4 h. After the infection, cells were washed and fixed with 4% paraformaldehyde-PBS, and permeabilized with 0.1% Triton X-100-PBS. Polymerized actin was detected by staining with tetramethyl rhodamine isothiocyanate-phalloidin (Molecular Probes-Invitrogen, Carlsbad, CA, USA). DNA from nuclei and bacteria were detected using TO-PRO-3 (Molecular Probes-Invitrogen, Carlsbad, CA, USA).

#### For immunofluorescence analysis

Treated cells were fixed and permeabilized and then were blocked with 1% bovine serum albumin (BSA). Subsequently, activated NF-κB and ERK1/2 were detected by incubating the cells with anti-phospho-p65 or anti-phospho-ERK1/2 antibodies (Cell signaling Technology, Danvers, MA, USA) as indicated by the manufacturer, followed by FITC-goat anti-rabbit IgG and FITC-goat anti-mouse IgG antibodies, respectively, (Thermo Fisher Scientific, Waltham MA, USA). Slides were mounted with VectaShield (Vector Laboratories, Burlingame, CA, USA) covered with glass coverslips and analyzed using a Leica Confocal Microscope TCS SP8 (Leica Microsystems, Wetzlar, Germany) and Leica LAS AF lite software. Maximal projections from about 30 sections of 0.4 μm are shown.

#### For kinetics of infection and immunoblot

Starved cells that had been cultured in 30 × 10 mm dishes (Corning, NY, USA) were washed and kept in DMEM without FBS and antibiotics. Cells were inoculated with the corresponding bacterial cultures at a MOI of 10 and incubated for 30 min, 1, 2, and 4 h. Mock conditions were cells that received only the interaction medium without bacteria. After the infection, cells were washed with washing buffer (20 mM Glycerol 3-phosphate and 20 mM NaF), scraped with 200 μl of lysis buffer (50 mM Tris-HCl, pH 7.4, 150 mM NaCl, 1 mM EDTA, 1% IGEPAL, 1% Sodium deoxycholate, 10 mM Na_4_P_2_O_7_, 10 mM NaF, 10 mM Na_3_VO_4_, protease inhibitor cocktail, Complete™) for 15 min on ice and centrifuged (21,255 × g, 15 min at 4°C). Proteins from supernatants were quantified by the Bradford method and analyzed by immunoblot. Equal amounts of proteins were loaded on 10% SDS-PAGE gel and transferred to the PVDF membrane. Membranes were reacted with antibodies against phospho-ERK1/2, total ERK1/2, phospho-IκB-α, total IκB-α, total p65, or phospho-p65 (Cell signaling Technology, Danvers, MA, USA) and actin (a gift from Dr. José Manuel Hernández, a monoclonal antibody produced at the CINVESTAV) followed by HRP-conjugated rabbit anti-mouse IgG2a antibody or HRP-conjugated goat anti-rabbit IgG antibody. Protein bands density was quantified using Image J, NIH Software.

### Co-stimulation assays

After the infection as mentioned above, cells were washed and incubated with fresh medium supplemented with 100 μg/ml of Gentamicin at 37°C for 1 h in a 5% CO_2_ incubator. After killing the bacteria, cells were washed and the medium was replaced with fresh DMEM with 10 ng/ml of either TNF-α or EGF (PeproTech, Inc., Rocky Hill, NJ, USA) for 10 min. Cells were collected and lysed as mentioned above. Proteins from supernatants were quantified by the Bradford method and analyzed by immunoblot as mentioned in the previous section.

### Determination of cytokine concentrations in epithelial cells

Pro-inflammatory cytokines levels (IL-8, TNF-α, IL-1β, IL-6, and anti-inflammatory IL-10) were simultaneously measured by flow cytometry using human inflammatory cytokines (CBA) kit (BD Cytometric Bead Array, Biosciences Pharmingen, San Diego, USA). All procedures were done following the manufacturer's instruction. The data acquisition was performed using a flow cytometer (BD LSR Fortessa™). Cytokines were quantitatively measured in culture supernatants from HT-29 cells inoculated with the different *E. coli* pathotypes or the non-pathogenic strain HB101 at a MOI of 10 during 30 min, 1, 2, or 4 h or treated with 100 ng/ml of lipopolysaccharide (LPS) from *E. coli* serotype 0128:B12 (Sigma-Aldrich, St Louis, Missouri, USA). The quantitative results were generated using the software FCAP Array™ version 3.0 (Soft Flow Hungary Ltd.,).

### Determination of cytokine concentrations in macrophage-like cells

After differentiation into THP-1 macrophage-like, cells were washed and RPMI-1640 without supplements was added and starved in the same medium for 24 h at 37°C in a 5% CO_2_ incubator. THP-1 cells were inoculated with the corresponding bacterial cultures at a MOI of 10 during 2 and 4 h or treated with 100 ng/ml of LPS during 4 h. Culture supernatants were collected and analyzed by flow cytometry using human inflammatory cytokine (CBA) kit as mentioned above.

### Inhibitors

The NF-κB pathway inhibitor (BAY 11-7082) and the ERK1/2 pathway inhibitor (PD98059) (PeproTech, Rocky Hill, NJ, USA) were stored in dimethyl sulfoxide (DMSO) at −20°C. Prior to the infection, the cells were incubated with either BAY 11-7082 (100 μM) or PD98059 (100 μM), or both for 1 h. The inhibitors were maintained during the infection period.

### Statistical analysis

Data represent means ± SEM. Data comparisons were performed with two-way ANOVA tests and *post-hoc* Tukey test or Student's *t*-test using GraphPad Software (La Jolla, CA, USA). Differences were considered significant at *p* ≤ 0.05.

## Results

### Reproduction of the phenotypic features induced by the different pathotypes in a standardized epithelial cell model

**Figure 1:**
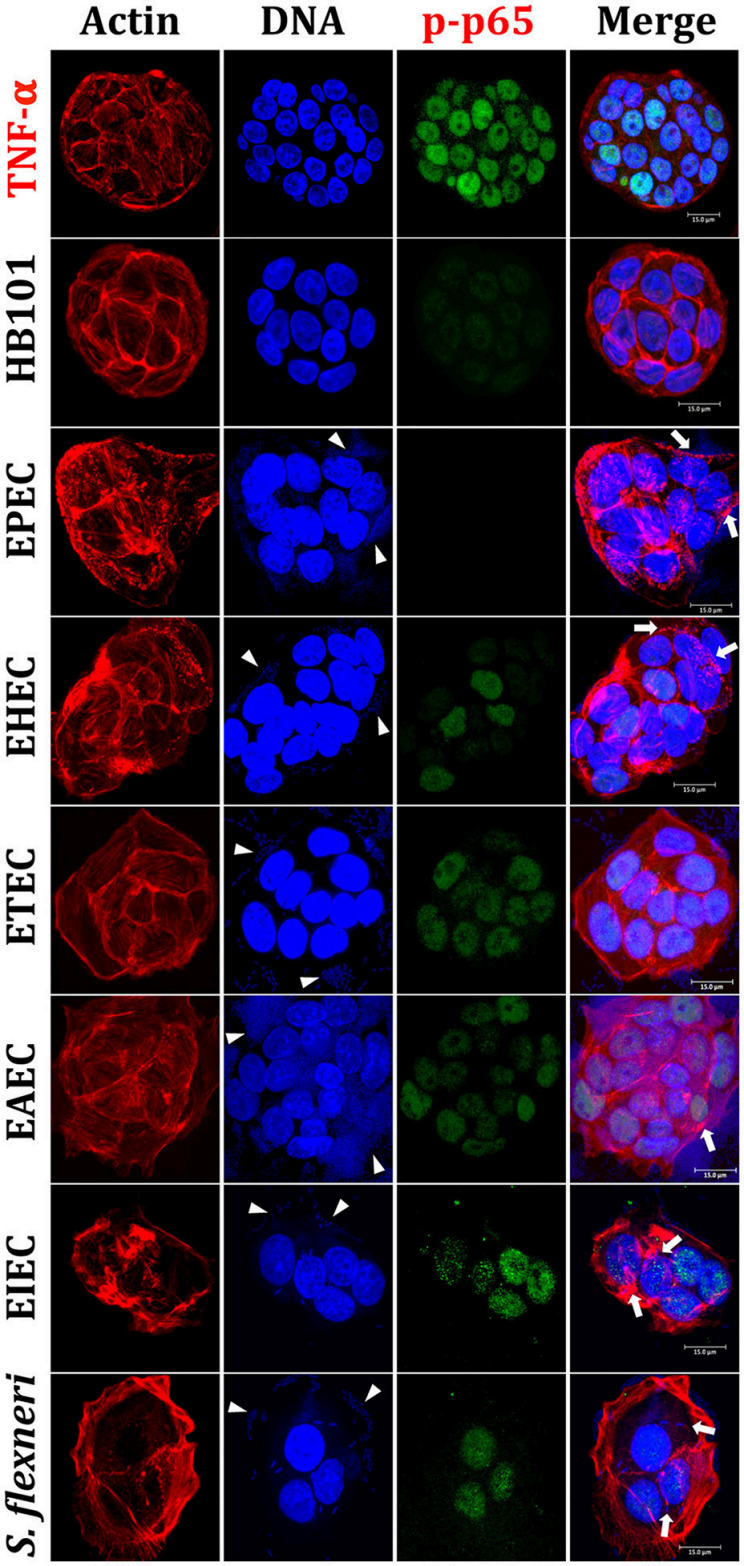
**Reproduction of the phenotypic features (adhesion, cytotoxicity and actin cytoskeleton rearrangements) induced by different *E. coli* pathotypes and nuclear translocation of NF-κB in HT-29 cells as intestinal model**. HT-29 cells were infected (MOI 10) during 4 h with the different *E. coli* pathotypes as indicated. As controls, cells were treated with *E. coli* HB101, a non-pathogenic strain, or with TNF-α, a NF-κB translocation inducer. After treatment, cells were washed, fixed and stained with rhodamine-phalloidin for actin filaments and TO-PRO-3 for DNA detection, while NF-κB translocation was detected by immunofluorescence with anti-phospho-NF-κB (phospho-p65) antibodies followed by FITC-goat anti-rabbit IgG antibodies. The preparations were analyzed and documented with a confocal microscope (63X). Arrowheads indicate the location of bacterial adhesion and arrows point out cytoskeleton rearrangements (pedestal formation induced by EPEC, EHEC), rounding cell (EAEC), and cell invasion (intracellular actin tails by EIEC and *S. flexneri*). Bar 15 μm.

**Figure 2:**
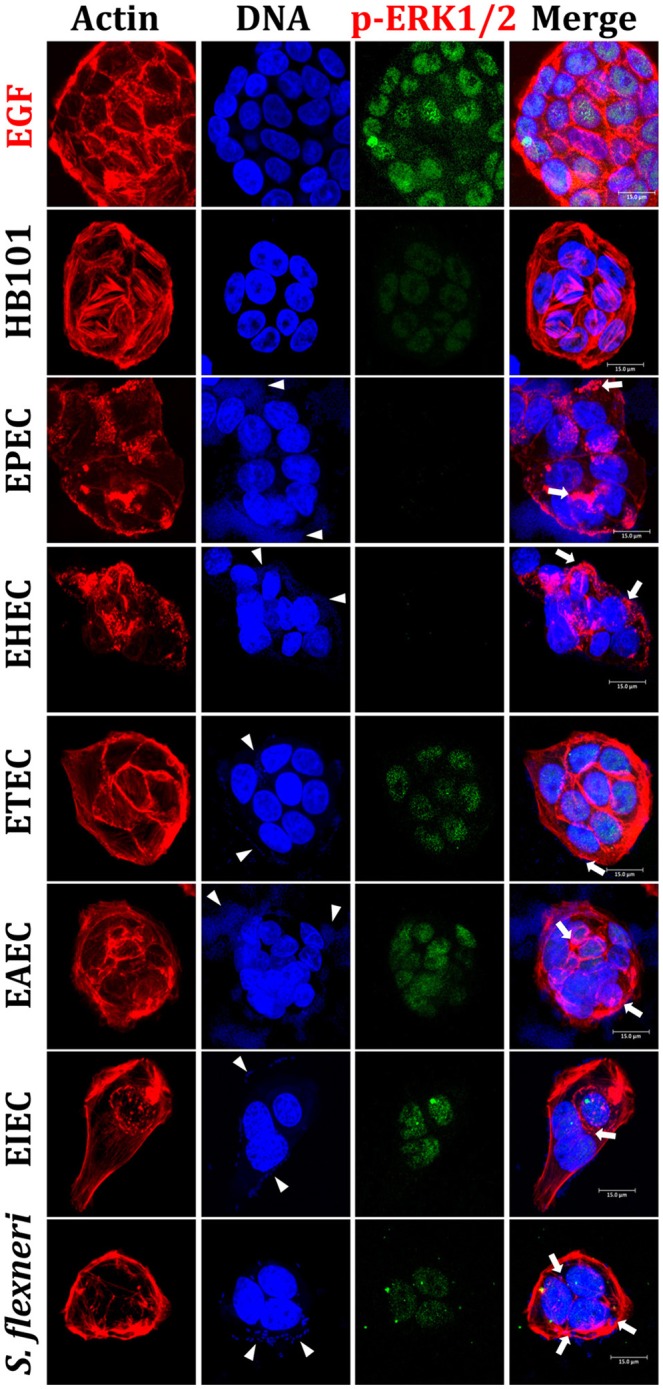
**Reproduction of the phenotypic features induced by different *E. coli* pathotypes and nuclear translocation of ERK1/2 in HT-29 cells**. HT-29 cells were infected (MOI 10) during 4 h with the different *E. coli* pathotypes as indicated. As controls, cells were treated with *E. coli* HB101, a non-pathogenic strain, or with EGF, an ERK1/2 translocation inducer. After treatment, cells were processed for confocal microscopy as indicated in Figure 1, but ERK1/2 translocation was detected by immunofluorescence using anti-phospho-ERK1/2 antibodies followed by FITC-goat anti-mouse IgG antibodies. Arrowheads indicate the location of bacterial adhesion and arrows point out cytoskeleton rearrangements (pedestal formation induced by EPEC, EHEC), rounding cell (EAEC), and cell invasion (intracellular actin tails by EIEC and *S. flexneri*). Bar 15 μm.

In order to evaluate and compare the inflammatory responses induced by each pathotype, we first standardized an intestinal epithelial cells (IECs) model, which could reproduce phenotypic features induced by each pathotype, such as adhesion, cytotoxicity, and actin cytoskeleton rearrangements. To do that, HT-29 cells were infected with the different pathotypes at different multiplicity of infection (MOI) and different infection times (data not shown). We found that 4 h of infection at a MOI of 10 were the optimal conditions to evaluate bacteria adhesion, cytotoxicity and actin cytoskeletal rearrangement and these conditions were reproducible for each pathotype. By using these conditions, HT-29 cells were infected with the different pathotypes and the infected cells were prepared for confocal microscopy. Mock cells were observed with a homogeneous morphology (data not shown), firmly adhered and extended cells forming compact colonies, showing their integral actin stress fibers as detected by F-actin assay (FAS). Cells treated with HB101, a non-pathogenic strain, were also observed with a normal morphology as the mock cells, without bacterial adhesion or actin stress fibers disruption (Figures 1, 2). In cells infected by EPEC, it was possible to observe the characteristic localized adherence pattern forming bacterial microcolonies, as detected by bacterial DNA using TO-PRO-3, and the accumulation of polymerized actin beneath the site of bacterial attachment to form a pedestal-like structure detected by FAS. In cells infected by EHEC, bacterial adhesion was observed but without localized adherence. Most of the adhered bacteria were observed forming pedestal-like structures. ETEC was unable to cause clear morphological changes in HT-29 cells but it was possible to detect randomly bacterial adhesion without actin cytoskeleton disruption. EAEC was able to adhere in the classic aggregative adherence pattern, with the typical stacked-brick binding pattern. Morphological changes were detected as discrete zones of discontinued actin stress fibers as well as irregular actin accumulation. On the other hand, the intracellular bacteria, EIEC, and *Shigella flexneri* (used as a positive control), caused the most evident morphological changes by inducing strong actin cytoskeleton damage. Bacteria were observed adhered to the plasma membrane and inside the cells and these intracellular bacteria were seen forming F-actin comet tails (Figures 1, 2). All these data allowed us to validate the HT-29 cells as an infection model for the induction of main phenotypic features caused by the diarrheagenic *E. coli* pathotypes evaluated.

### Differential nuclear translocation of NF-κB and ERK1/2 induced by the diarrheagenic pathotypes

Once validated the infection model, we evaluated next the nuclear NF-κB translocation in IECs infected by the different pathotypes through phosphorylated p65 translocation. HT-29 cells were infected for 4 h with the different pathotypes (MOI of 10) or treated with TNF-α (inducer control). Infected cells were stained with rhodamine-phalloidin and TO-PRO-3 to detect F-actin and DNA from bacteria and eukaryotic cells, respectively. Mock cells were observed with a homogeneous morphology without green signal into nuclei indicating no phospho-p65 translocation (data not shown). Phospho-p65 translocation induced by TNF-α was clearly detected by the anti-phospho-p65 as a green color inside nuclei and a cyan color in the merge images showing co-localization of DNA (blue) and phospho-p65 (green) (Figure 1). HB101 was able to cause a very slightly p65 translocation. Interestingly, at 4 h of infection EPEC did not cause phospho-p65 nuclear translocation since phosphoprotein was undetectable into nuclei. EHEC induced slight phospho-p65 nuclear translocation at 4 h of infection and DNA/phospho-p65 co-localization was less evident; interestingly, this translocation was lower where enriched pedestal-like structure zones were localized (Figure 1). On the other hand, ETEC and EAEC showed a clear homogeneous phospho-p65 nuclear translocation and in both cases the DNA/phospho-p65 co-localization was evident in all cell nuclei. In cells infected by intracellular bacteria (EIEC and *S. flexneri*), it was also possible to detect phospho-p65 translocation inside nuclei and in both cases the DNA/phospho-p65 co-localization was also evident; however, both effects were higher in cells infected with EIEC (Figure 1).

By using a similar approach, we evaluated ERK1/2 nuclear (phospho-ERK1/2) translocation in IECs infected by the different pathotypes. HT-29 cells were infected for 4 h with the different pathotypes (MOI of 10) or treated with EGF (inducer control). Again, in EGF-treated cells, phospho-ERK1/2 translocation was clearly detected by the anti-phospho-ERK1/2 as green color inside nuclei and cyan color in the merge images showing co-localization of DNA (blue) and phospho-ERK1/2 (green) (Figure 2). Contrastingly, mock cells did not show phospho-ERK1/2 translocation and no green signal was detected into nuclei (data not shown). As expected, HB101 was unable to cause ERK1/2 translocation since phospho-ERK1/2 was almost undetectable. Interestingly, neither EPEC nor EHEC were able to induce phospho-ERK1/2 translocation inside nuclei and in both cases this protein was undetectable in the infected cells, despite the strong cytoskeletal disruption caused by both pathotypes (Figure 2). ETEC and EAEC caused phospho-ERK1/2 nuclear translocation, which were more evident in cells infected with ETEC. Thereby, the DNA/phospho-ERK1/2 co-localization inside nuclei was more evident in these cells infected with ETEC; despite EAEC caused more cell damage than ETEC. On the other hand, both intracellular bacteria, EIEC and *S. flexneri* induced phospho-ERK1/2 nuclear translocation and it was stronger in cells infected by EIEC than by *S. flexneri*; although there are more intracellular bacteria in cells infected by *S. flexneri* than by EIEC (Figure 2).

All these data indicate than the different pathogenic schemes and virulence factors of pathotypes caused differential signal transduction for inducing NF-κB and ERK1/2 nuclear translocation and suggested that this signal transduction could be manipulated during the infection by these different pathotypes. For instance, EPEC does not cause phospho-p65 or phospho-ERK1/2 nuclear translocation while EHEC does cause phospho-p65, but not phospho-ERK1/2, nuclear translocation. Interestingly, a lower nuclear factors translocation was detected in cells being infected by extracellular bacteria, except for those infected by ETEC (where the morphological damage were undetectable).

### Pathotypes are able to differentially manipulate the p65 (NF-κB) activation

**Figure 3:**
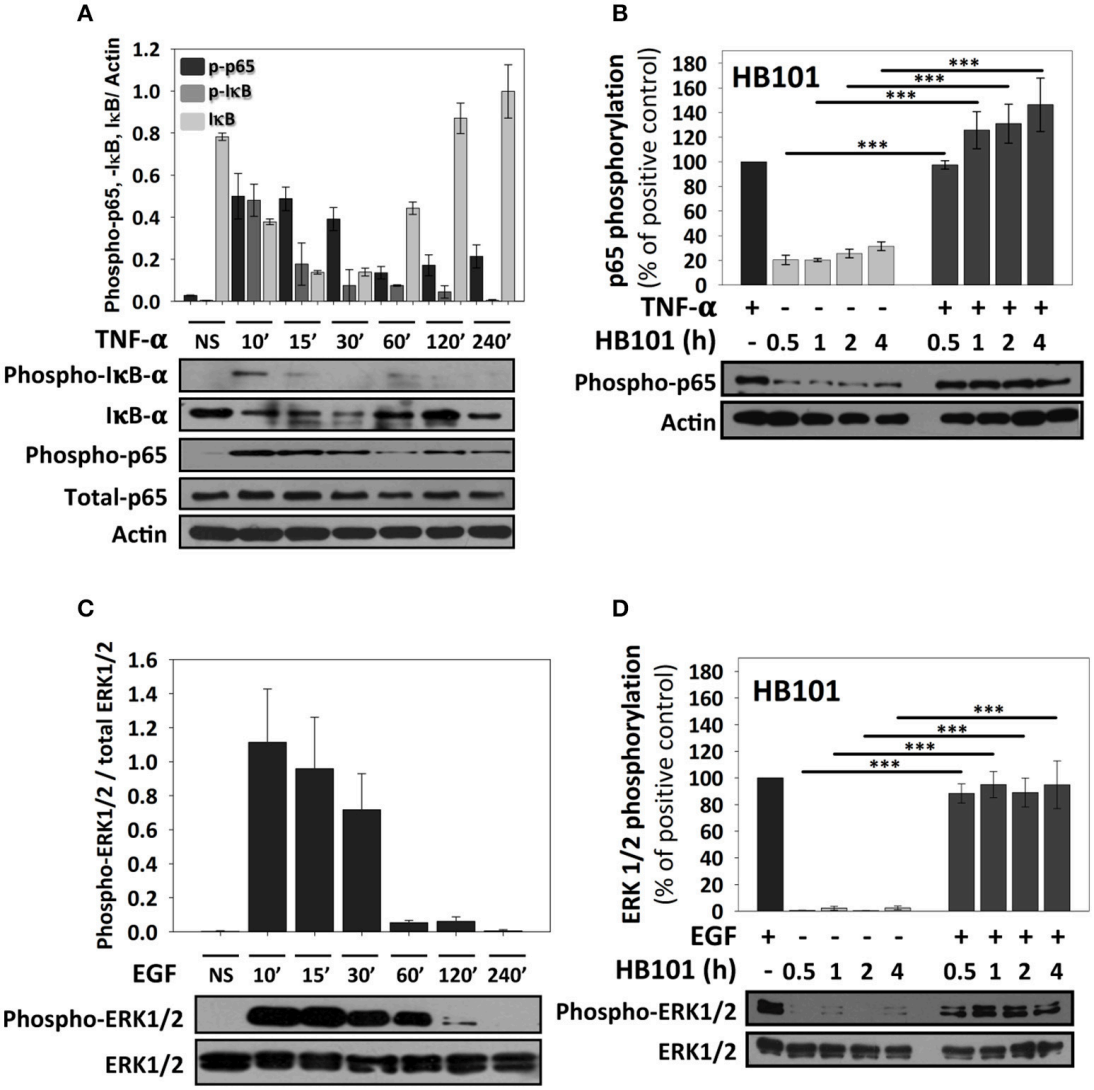
**TNF-α and EGF induce a strong activation of NF-κB and ERK1/2 signaling pathways respectively, but not a non-pathogenic strain, in HT-29 cells. (A,B)** Effects of TNF-α and EGF on epithelial cells. HT-29 cells were stimulated with TNF-α or EGF (10 ng/ml) during 10, 15, 30, 60, 120, and 240 min. **(C,D)** Effects of *E. coli* HB101 on epithelial cells and its co-stimulation with either TNF-α or EGF. HT-29 cells were inoculated with the non-pathogenic strain *E. coli* HB101 at a MOI of 10 for 0.5, 1, 2, and 4 h and divided into two groups. The first group was directly processed after *E. coli* HB101 inoculation. In the second group, after incubation, cells were treated with Gentamicin (100 μg/ml) to kill bacteria for 1 h prior to adding TNF-α **(C)** or EGF **(D)** (10 ng/ml) for 10 min. Cellular extracts were analyzed by Western Blot using primary specific antibodies for NF-κB (phospho-p65, phospho-IκB-α and IκB-α) or ERK1/2 (phospho-ERK1/2 and total ERK1/2) and actin, following by HRP-goat anti-rabbit IgG or HRP-rabbit anti-mouse IgG2a. Uninfected cells stimulated with TNF-α or EGF represent 100% of activation for NF-κB **(C)** or ERK1/2 **(D)** (as positive controls). All proteins evaluated were normalized with actin. Densitometry analysis shows the means ± SEM for at least three independent experiments. ^***^p < 0.001 comparing stimulated versus co-stimulated at each time point, using two-way ANOVA test and *post-hoc* Tukey test.

Since the analyses of confocal microscopy were performed at 4 h of infection with the different pathotypes and to further understand why these bacteria produce these differential responses, we performed kinetics of infection using the different pathotypes and detecting NF-κB activation through detection of p65 phosphorylation using immunoblot with anti-phospho-p65 antibodies. At the same time to understand any modulation of NF-κB signal transduction pathway by the different pathotypes, the cells were co-stimulated with TNF-α after the induction of p65 phosphorylation by the infection. Thus, we first standardized the optimal time of p65 phosphorylation by TNF-α and its correlation with IκB-α phosphorylation and subsequent degradation (detected by antibodies against phosphorylated IκB-α and total IκB-α). To do that, HT-29 cells were incubated with TNF-α (10 ng/ml) at different times (10, 15, 30, 60, 120, and 240 min) and the phosphorylated proteins (IκB-α and p65) were detected by immunoblot. In untreated cells total IκB-α is detected as a thick protein band (no degradation) and there are no bands detected by anti-phospho-IκB-α or anti-phospho-p65 antibodies. At 10 min of TNF-α treatment, it was possible to observe phosphorylation of IκB-α, which coincided with IκB-α degradation (in about 50% respect to untreated cells) and, as expected, a clear band of phosphorylated p65 was detected that decreased at 60, 120, and 240 min (Figure 3A). Thus, at 15 min, the bands of phosphorylated IκB-α dramatically decreased (about 40%) mainly because total IκB-α band was also decreasing. Interestingly, phosphorylated p65 was unchanged at 15 and 30 min. However, at 60 min and with next treatment times, total IκB-α started again to increase (Figure 3A). Total p65 protein band did not change in all times tested. Once validated the NF-κB activation, for subsequent co-stimulation experiments, 10 min of stimulation with TNF-α was used. We initially tested p65 phosphorylation induced by the non-pathogenic *E. coli* HB101 at different interaction times (0.5, 1, 2, and 4 h). HB101 was unable to induce p65 phosphorylation at any time tested and only slight bands were detected, which represented about 20–30% of p65 phosphorylation induced by TNF-α (positive control), which was arbitrarily defined as 100% of phosphorylation. Interestingly, co-stimulation with TNF-α after HB101 infection clearly induced p65 phosphorylation at 30 min of infection and caused an increase in 100%, similar to the TNF-α treatment alone. The phosphorylated p65 levels increased with the infection time beyond 100%, suggesting a synergistic effect between HB101 and TNF-α induction (Figure 3B).

**Figure 4:**
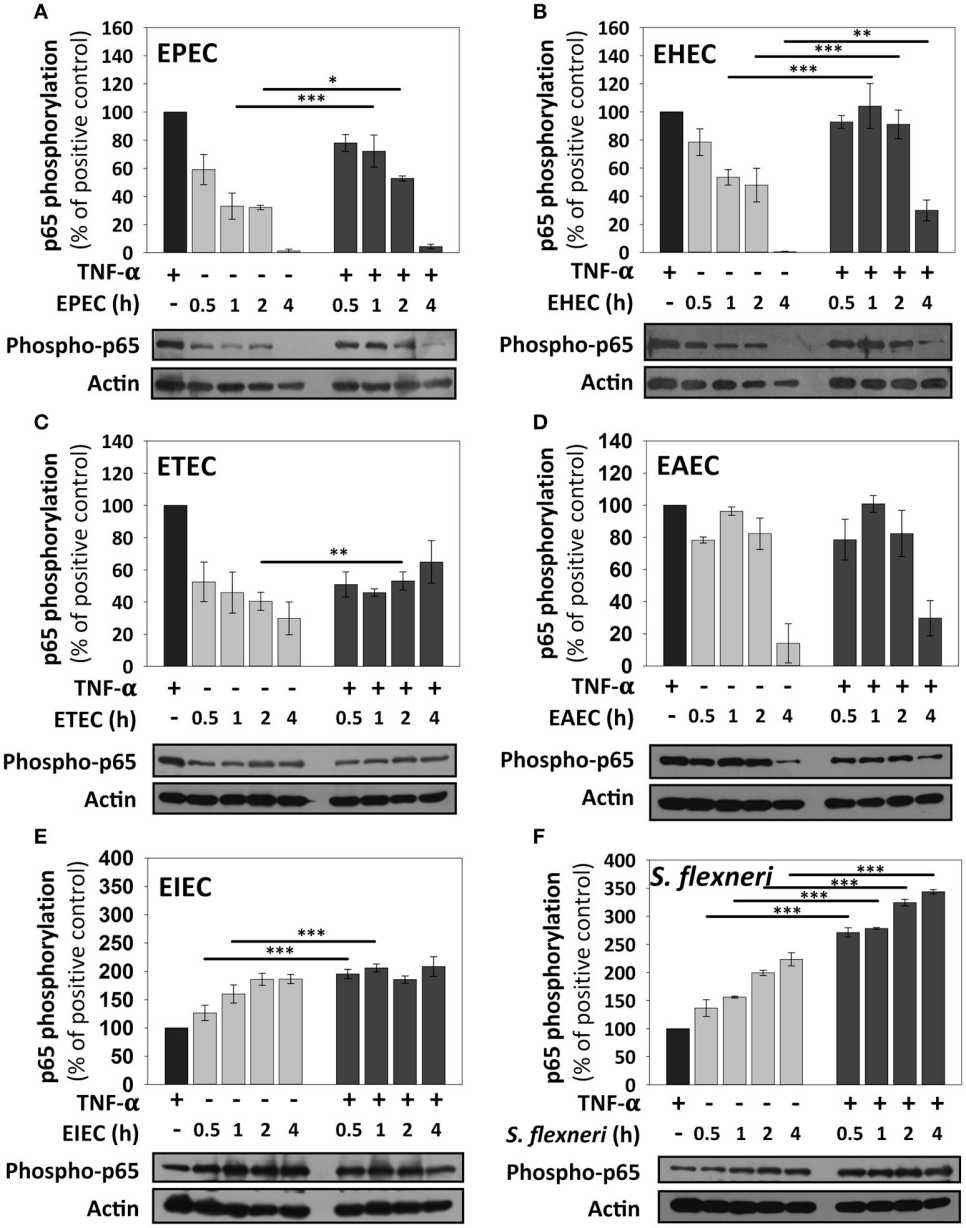
**NF-κB stimulation by the different *E. coli* pathotypes on HT-29 cells and its co-stimulation with TNF-α**. HT-29 cells were infected with the different *E. coli* pathotypes, EPEC **(A)**, EHEC **(B)**, ETEC **(C)**, EAEC **(D)**, EIEC **(E)**, and *S. flexneri* **(F)** at a MOI of 10 for 0.5, 1, 2, and 4 h and divided into two groups. The first group was directly processed after the infection. In the second group, after incubation, cells were treated with Gentamicin (100 μg/ml) to kill bacteria for 1 h prior to adding TNF-α (10 ng/ml) during 10 min. Cellular extracts were analyzed by Western Blot using primary specific antibodies for NF-κB (phospho-p65) and actin, following by HRP-goat anti-rabbit IgG antibody. Uninfected cells stimulated with TNF-α (as a positive control). All proteins evaluated were normalized with actin. Densitometry analysis shows the means ± SEM for at least three independent experiments. ^*^p < 0.05, ^**^p < 0.01, ^***^p < 0.001 comparing stimulated versus co-stimulated at each time point, using two-way ANOVA test and *post-hoc* Tukey test. For easy comparison, gray bars indicate infected cells and dark gray those co-stimulated cells.

By using this standardized approach, we compared kinetics of p65 phosphorylation induced by the different pathotypes as well as the effects of the co-stimulation with TNF-α. HT-29 cells infected with EPEC, which had shown no p65 phosphorylation at 4 h in confocal microscopy experiments, showed that indeed there was not p65 phosphorylation band as detected by immunoblot at 4 h of infection. However, EPEC infection caused p65 phosphorylation at 0.5 h of infection and it represented 60% of the response induced by TNF-α alone. This response decreased to 30% at 1 and 2 h of infection. Interestingly, co-stimulation with TNF-α after 0.5 h of EPEC infection increased p65 phosphorylation, but this increase (75%) was lower that the induced by TNF-α treatment alone. Moreover, the co-stimulation after 1 and 2 h of EPEC infection was decreasing with the time to 70 and 50%, respectively, until be undetectable at 4 h of infection (Figure 4A). EHEC caused p65 phosphorylation and the increase of the phosphorylated protein band was about 80%, compared with those induced by the positive control (TNF-α) at 0.5 h of infection; this percentage decreased to about 50% at 1 and 2 h of infection until disappearing at 4 h. Unlike EPEC, cells co-stimulated with TNF-α after 0.5, 1, and 2 h of EHEC infection reached about 100% of p65 phosphorylation, similar to the positive TNF-α control. Co-stimulation after 4 h of infection, only decreased to 30% the p65 phosphorylation; unlike EPEC that caused a full reduction of p65 phosphorylation after co-stimulation (Figure 4B). Interestingly, ETEC caused a sustained p65 phosphorylation at 0.5, 1, 2, and 4 h of infection with an increase of about 30–50%. In cells co-stimulated after the four different infection times the increase of p65 phosphorylation only reached percentages of around 50–60% (Figure 4C). From all these extracellular bacteria, EAEC was the highest p65 phosphorylation inducer with percentages around 80–95% and this response was sustained at 0.5, 1, and 2 h of infection until decreasing to 10% at 4 h of infection. Remarkably, co-stimulation with TNF-α after 0.5, 1, 2, and 4 h of infection by EAEC, induced levels of p65 phosphorylation very similar to those of cells infected without co-stimulation (Figure 4D). On the other hand, the intracellular bacteria responded quite different regarding to NF-κB activation measured as p65 phosphorylation. The EIEC infection caused a strong p65 phosphorylation at 0.5 h of infection, which was higher (125%) than those responses induced by the positive TNF-α control. This response increased (160%) at 1 h of infection and reached an increase of 185% at 2 and 4 h of infection with respect to those induced by TNF-α alone. Interestingly, co-stimulation after infection at all the times tested caused a two-fold increase in p65 phosphorylation (about 200%) that those induced by TNF-α alone (Figure 4E). *S. flexneri* caused an increase of p65 phosphorylation in a time-dependent way with percentages higher than TNF-α alone (135, 160, 200, and 220% at 0.5, 1, 2, and 4 h, respectively). Moreover, co-stimulation with TNF-α after *S. flexneri* infection caused the highest increase of p65 phosphorylation. At 0.5 and 1 h of infection, the co-stimulation increased levels of p65 phosphorylation to 270% with respect to TNF-α alone and clearly higher than those values reached by *S. flexneri* infection alone. While, at 2 and 4 h of infection the co-stimulation increased the levels of p65 phosphorylation to 320 and 340% respect to TNF-α alone (Figure 4F).

### ERK1/2 activation is also differentially manipulated by the different pathotypes

ERK1/2 activation was initially standardized by using EGF induction at different times and detecting ERK1/2 phosphorylation as previously described (Jijon et al., 2012) and then validated after HB101 infection at 0.5, 1, 2, and 4 h. EGF induced ERK1/2 phosphorylation at 10, 15, and 30 min of interaction with HT-29 cells but not at 120 and 240 min of interaction (Figure 3C). Thereby, in subsequent experiments of co-stimulation and as a positive control, EGF was used at 10 ng/ml for 10 min. HB101 infection did not cause induction of ERK1/2 phosphorylation at any time tested. Whereas, co-stimulation with EGF after HB101 infection caused an increment of ERK1/2 phosphorylation at 100% at the different infection times tested, similar to those levels induced by the positive EGF control (Figure 3D).

**Figure 5:**
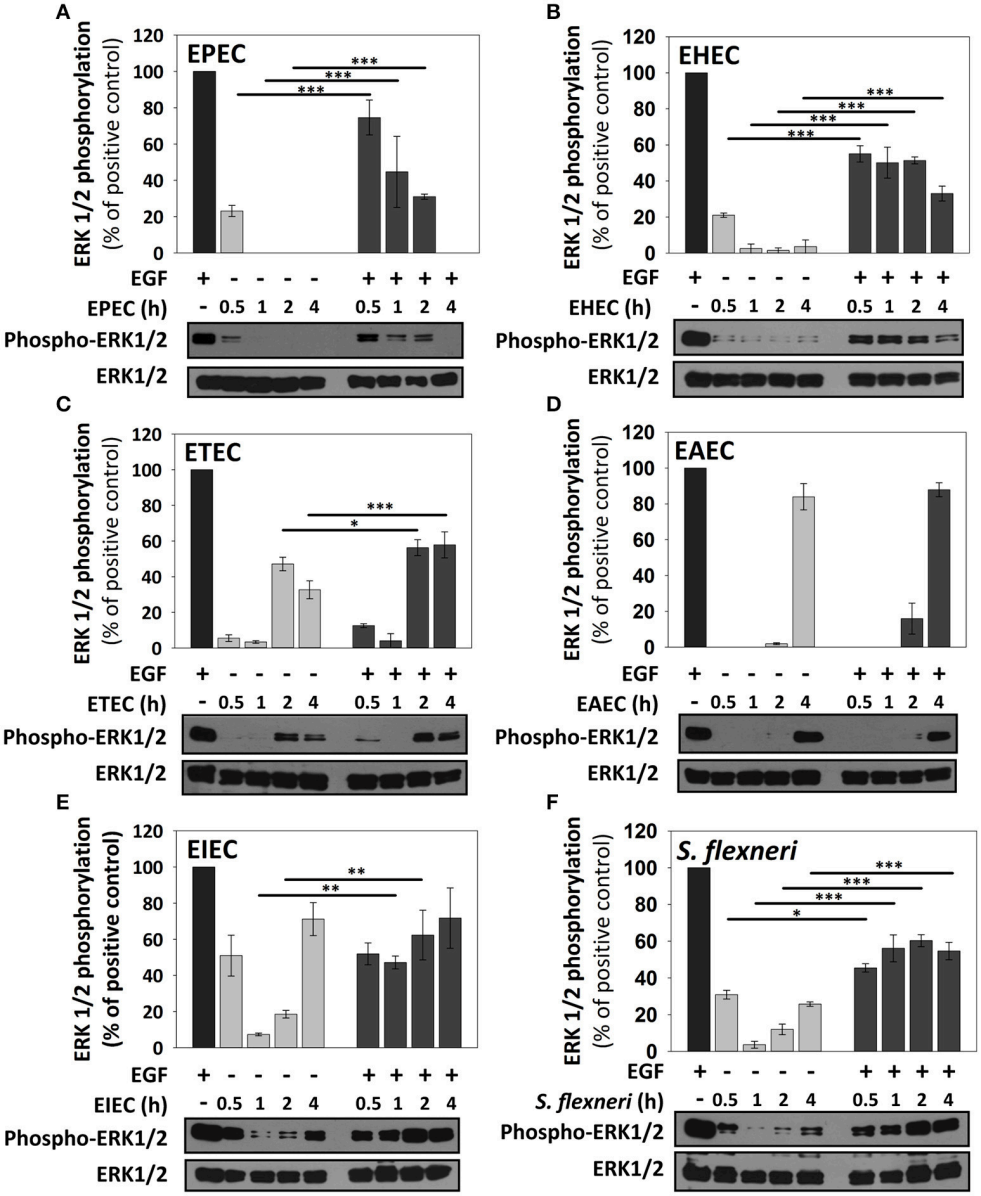
**ERK1/2 stimulation by the different *E. coli* pathotypes on HT-29 cells and its co-stimulation with EGF**. HT-29 cells were infected with the different *E. coli* pathotypes, EPEC **(A)**, EHEC **(B)**, ETEC **(C)**, EAEC **(D)**, EIEC **(E)**, and *S. flexneri* **(F)** at a MOI of 10 for 0.5, 1, 2, and 4 h and divided into two groups. The first group was directly processed after the infection. In the second group, after incubation, cells were treated with Gentamicin (100 μg/ml) to kill bacteria for 1 h prior to adding EGF (10 ng/ml) during 10 min. Cellular extracts were analyzed by Western Blot using primary specific antibodies for ERK1/2 and phosphorylated-ERK1/2, following by HRP-rabbit anti-mouse IgG2a antibody. Uninfected cells stimulated with EGF (as a positive control). ERK1/2 activation values were normalized with total ERK1/2. Densitometry analysis shows the means ± SEM for at least three independent experiments. ^*^p < 0.05, ^**^p < 0.01, ^***^p < 0.001 comparing stimulated versus co-stimulated at each time point, using two-way ANOVA test and *post-hoc* Tukey test. For easy comparison, gray bars indicate infected cells and dark gray those co-stimulated cells.

EPEC only caused a modest increment of ERK1/2 phosphorylation at 0.5 h of infection, but no other infection time points, representing about 25% of levels induced by the positive EGF control. Interestingly, co-stimulation with EGF after EPEC infection decreased ERK1/2 phosphorylation (75, 45, 30, and 0% at 0.5, 1, 2, and 4 h) in comparison to the co-stimulation after HB101 infection (100% at all tested times; Figure 5A). EHEC infection also caused an increase of ERK1/2 phosphorylation in only 20% at 0.5 h of infection and co-stimulation with EGF after EHEC infection only increased 50–55% at 0.5, 1, and 2 h of infection and 30% at 4 h in comparison with EGF alone (100%; Figure 5B). Unlike A/E pathogens as EPEC and EHEC, the specific pathogenic scheme of ETEC and EAEC increased ERK1/2 phosphorylation at late time points (2 h and/or 4 h of infection). ETEC infection increased ERK1/2 phosphorylation at 2 h (45%) and 4 h (35%) of infection. Interestingly, co-stimulation with EGF following ETEC infection blocked ERK1/2 phosphorylation at 0.5 and 1 h of infection, while at 2 and 4 h only reached 55% of the positive control (Figure 5C). On the other hand, EAEC infection caused a strong increase of ERK1/2 phosphorylation only at 4 h (85%). Remarkably, co-stimulation with EGF after EAEC infection did not cause an increase in ERK1/2 phosphorylation at 0.5, 1, and 2 h, but at 4 h by increasing similar to ETEC infection alone at 4 h (85%; Figure 5D). In the case of intracellular bacteria, the kinetics of ERK1/2 activation was different that those induced by extracellular bacteria. Thus, EIEC induced an increase of 50% of ERK1/2 phosphorylation at 0.5 h and lower than 20% at 1 and 2 h but 75% at 4 h of infection, while co-stimulation with EGF after infection, only caused an increase in about 50% at 0.5 and 1 h, and 60 and 70% at 2 h and 4 h, respectively (Figure 5E). *S. flexneri* infection only caused an increase of 30, 10, and 25% of ERK1/2 phosphorylation at 0.5, 2, and 4 h of infection, respectively. Co-stimulation with EGF after *S. flexneri* infection did not cause 100% of ERK1/2 phosphorylation, but instead caused about 50% at all the times tested (Figure 5F). Thus, lowest responses at 1 and 2 h of infection with the intracellular bacteria were recovered to 50% by co-stimulating with EGF but at no time tested the EGF co-stimulation after infection reached a 100%.

### Each pathotype induces a particular profile of IL-8 and TNF-α secretion

**Figure 6:**
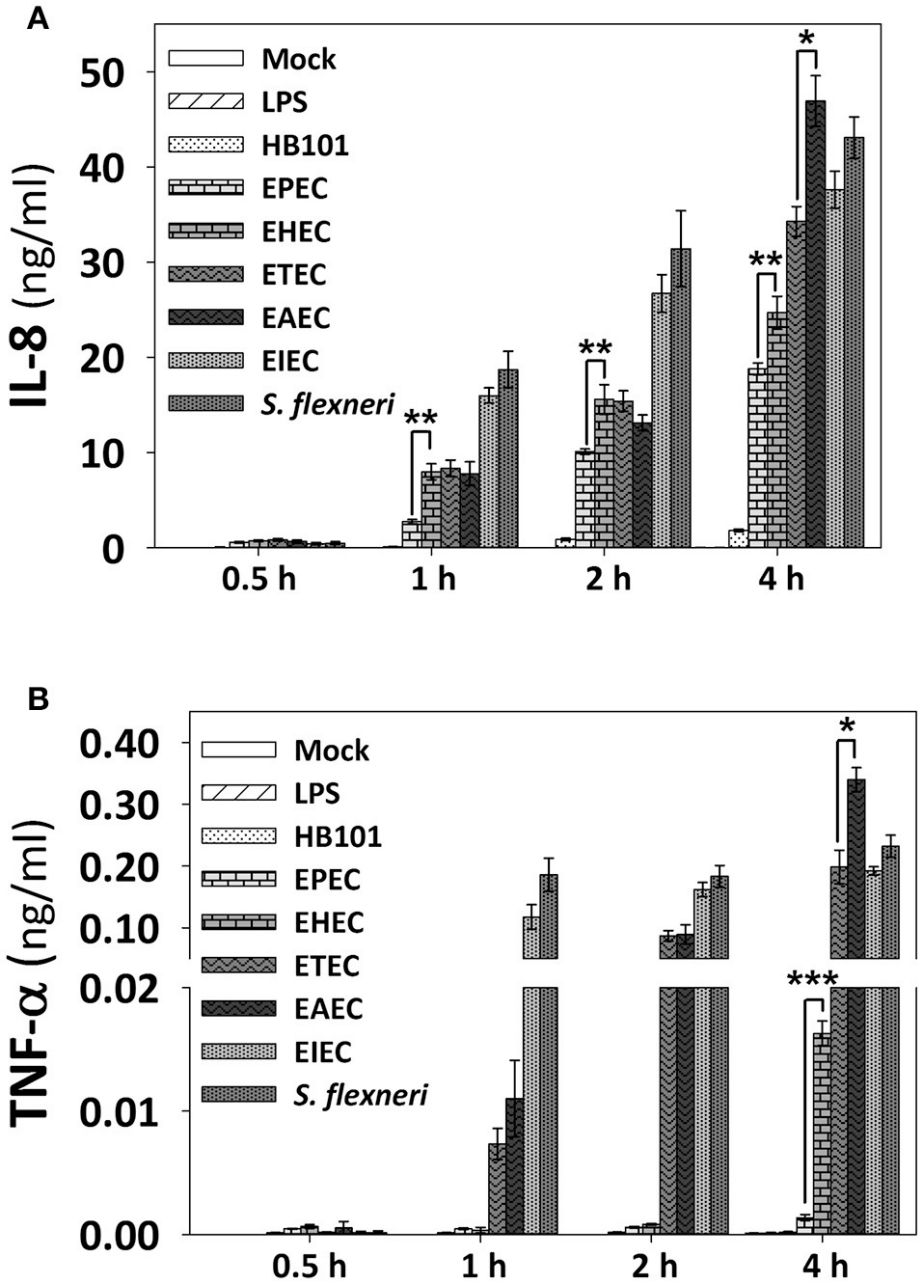
**IL-8 and TNF-α secretion induced by the different *E. coli* pathotypes in HT-29 cell model**. HT-29 cells were infected with the different *E. coli* pathotypes (MOI 10) during 0.5, 1, 2, or 4 h. After the infection, cell supernatants were removed and analyzed using Human inflammatory cytokines CBA kit (BD Biosciences). Data were acquired on a BD FACS Fortessa flow cytometer. All IL-8 **(A)** and TNF-α **(B)** data are means ± SEM of the three independent experiments. ^*^p < 0.05, ^**^p < 0.01, ^***^p < 0.001 comparing each pathotype by group, using Student's *t*-test.

In order to know the consequences of NF-κB and ERK1/2 activation induced by the pathotypes in term of cytokines secretion, we measured the cytokine secretion in the cell culture media after infection with the different pathotypes (MOI 10) at 0.5, 1, 2, and 4 h of infection. After the infection, cell supernatants were removed and analyzed using the human inflammatory cytokines CBA kit and flow cytometry. Even though this kit can detect six cytokines (IL-8, TNF-α, IL-1β, IL-6, IL-12p70, and IL-10), we were able to detect only IL-8 and TNF-α in HT-29 cells. Both A/E pathogens, EPEC and EHEC infection increased the secretion of IL-8 in a time-dependent manner, but EPEC caused a significantly lower increase of IL-8 secretion; 0.6, 2.7, 10, and 19 ng/ml at 0.5, 1, 2, and 4 h of infection, respectively, while EHEC infection caused increases in 0.7, 8, 15.5, and 24.6 ng/ml at 0.5, 1, 2, and 4 h of infection (Figure 6A). Interestingly, EPEC and EHEC infection induced low secretion of TNF-α at 0.5, 1, and 2 h post-infection with values around 0.0005 ng/ml. However, at 4 h of infection EHEC caused a sudden increase of TNF-α secretion in 0.0163 ng/ml vs. those induced by EPEC, 0.0013 ng/ml (around 10 times; Figure 6B). ETEC and EAEC infection caused similar IL-8 secretion at 0.5 h (around 0.7 ng/ml), 1 h (around 8 ng/ml), and 2 h (around 14 ng/ml), but at 4 h of infection, EAEC caused a significant increase of IL-8 secretion than ETEC (47 vs. 34.2 ng/ml; Figure 6A). Similarly, ETEC and EAEC only caused a significant increase of TNF-α secretion at 2 h with the same values (0.08 ng/ml). At 4 h of infection, the response was significantly different; EAEC caused a TNF-α secretion of 0.33 ng/ml while ETEC infection caused a secretion of 0.2 ng/ml of TNF-α (Figure 6B). Unlike the extracellular bacteria, the intracellular bacteria induced similar secretion of IL-8 and TNF-α. Both EIEC and *S. flexneri* caused increases in IL-8 secretion in a time-dependent manner; around 0.4 ng/ml at 0.5 h of infection, around 17 ng/ml at 1 h, around 28 ng/ml at 2 h, and around 42 ng/ml at 4 h of infection (Figure 6A). Similarly, TNF-α secretion was also analogous between the two intracellular strains with no response at 0.5 h of infection but at 1, 2, and 4 h the responses ranging 0.12 and 0.22 ng/ml, without a statistical difference (Figure 6B).

In summary, EPEC is able to efficiently inhibit IL-8 secretion in comparison with the other pathotypes at all the times tested. In a lower degree, EHEC also inhibited IL-8 secretion at all the times tested in comparison with the intracellular bacteria, and at 4 h of infection compared with the other pathotypes (except for EPEC). At 4 h of infection, EAEC was the higher IL-8 secretion inducer and slightly higher than those induced by *S. flexneri*. At 1 and 2 h of infection clearly the intracellular bacteria were better IL-8 secretion inducer than the extracellular bacteria (Figure 6A). Regarding TNF-α, EPEC did not induce TNF-α secretion at any time tested while EHEC only induced a modest response at 4 h of infection. ETEC induced a modest response at 1 h of infection, but this response increased to values similar to those induced by the intracellular bacteria, and for this latter group, the values were similar at 1, 2, and 4 h of infection. Interestingly, the higher inducer of TNF-α secretion was again EAEC (Figure 6B).

**Figure 7:**
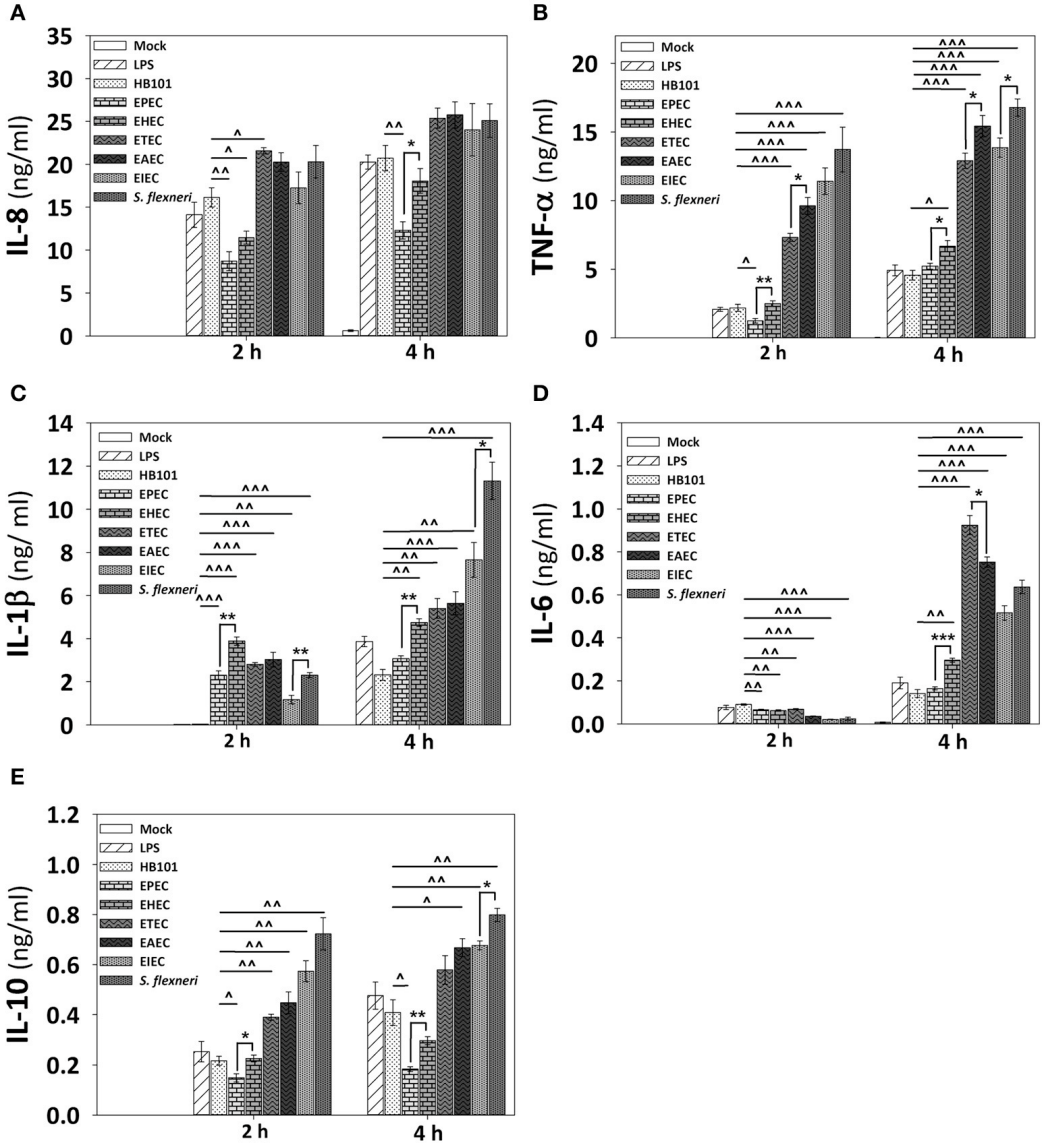
**Infection of human macrophages THP-1 cell line with the different *E. coli* pathotypes induce a differential cytokine secretion of IL-8, IL-1β, TNF-α, IL-6, and IL-10**. THP-1 cells were differentiated into macrophages-like cells using PMA at a concentration of 10 ng/mL during 48 h. After differentiation, adherent cells were infected with the different *E. coli* pathotypes (MOI 10) during 2 or 4 h. After infection, cell supernatants were removed and analyzed using Human inflammatory cytokines CBA kit. Data were acquired on a BD™ FACS Fortessa flow cytometer. Graphs represent cytokine secretion of IL-8 **(A)** and TNF-α **(B)**, as IL-1β **(C)**, IL-6 **(D)**, and IL-10 **(E)**. All data are means ± SEM of the three independent experiments. For statistical comparison, the pathotypes were grouped according to their way of interacting with the host cell as in Figure 6: ^*^p < 0.05, ^**^p < 0.01, ^***^p < 0.001 comparing each pathotype group or ^∧^*p* < 0.05, ^∧∧^*p* < 0.01, ^∧∧∧^*p* < 0.001 comparing each pathotype versus non-pathogenic *E. coli* HB101, using Student's *t*-test.

Since HT-29 cells appeared not to secrete the whole set of cytokines, which are detected by the human inflammatory cytokines CBA kit, and in order to investigate if cytokines response could have a similar profile between epithelial cells and macrophages regarding the secretion of IL-8 and TNF-α, we tested the detection of cytokines in PMA-differentiated THP-1 cells infected with all pathotypes at a MOI of 10 during 2 and 4 h. Interestingly, the profile of IL-8 secretion detected in THP-1 macrophage-like cells was very similar to those secreted by HT-29 epithelial cells; i.e., EPEC and EHEC pathotypes induced the lower levels of IL-8 secretion (12.3 and 18 ng/ml at 4 h post-infection vs. 19 and 24.6 ng/ml in HT-29 cells). These values were even lower that those induced by LPS or *E. coli* HB101 (Figure 7A). Remarkably, the profile of TNF-α secretion by THP-1 macrophages was also similar to those of HT-29 cells (with the exception of EHEC infection) but macrophages were more efficient secreting TNF-α than HT-29 cells; i.e., EHEC and mainly EPEC induced the lowest levels of TNF-α secretion in comparison with the other pathotypes, as the infected HT-29 cells, but up to1000 fold more TNF-α secretion than those secreted by HT-29 cells (6.7 and 5.2 ng/ml vs. 0.0163 and 0.0013 ng/ml; Figure 7B). These data suggest similar mechanisms of induction in both cell lines, which can be caused by damping extracellular receptors or intracellular signaling pathways. Additionally, unlike epithelial cells, THP-1 macrophage-like cells infected with the different pathotypes were also able to secrete IL-1β, IL-6, and IL-10 (Figures 7C–E). In general, EPEC and EHEC induced the lower secretion of these cytokines in comparison to the other pathotypes, while the intracellular bacteria induced the higher secretion of the cytokines. Interestingly, EAEC and mainly ETEC induced the highest levels of IL-6 at 4 h post-infection. These data suggest that the different *E. coli* pathotypes also modulate the cytokines secretion in macrophages.

### NF-κB activation is the main contributor of the IL-8 and TNF-α secretion responses

**Figure 8:**
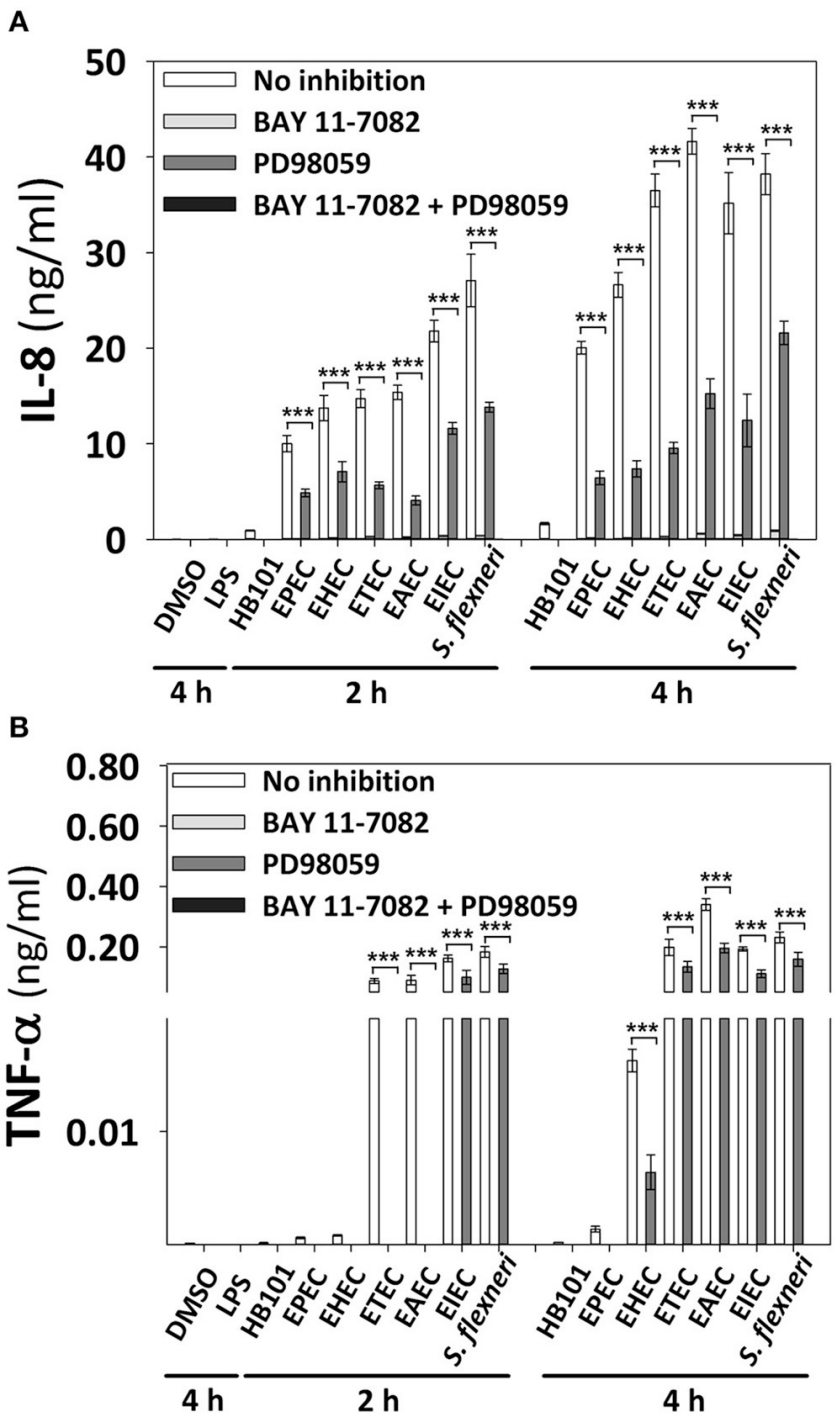
**Effect of the inhibitors of NF-κB and ERK1/2 in the cytokine secretion induced by the different *E. coli* pathotypes**. HT-29 cells were pretreated with inhibitors of NF-κB (BAY 11-7082) or ERK1/2 (PD98059) pathways or both, during 1 h at a concentration of 100 μM. After treatment, cells were carefully washed and infected with the different *E. coli* pathotypes (MOI 10) during 2 and 4 h. After the infection, the supernatants were removed and analyzed using Human inflammatory cytokines CBA kit. Data were acquired on a BD LSR Fortessa™ FACS flow cytometer. Graphs represent cytokine secretion of IL-8 **(A)** and TNF-α **(B)**. All data are means ± SEM of the three independent experiments. ^***^p < 0.001 comparing infected versus pretreated with inhibitors, using two-way ANOVA test and *post-hoc* Tukey test.

In order to identify the influence of signaling pathways (NF-κB or ERK1/2) inducing the secretion of epithelial cytokines upon different pathotypes infection, we used specific inhibitors. HT-29 cells were preincubated for 1 h with inhibitors of NF-κB (BAY 11-7082) or ERK1/2 (PD98059) single or combined, and then cells were infected with the different pathotypes for 2 or 4 h. Supernatants from infected cells were analyzed for IL-8 and TNF-α secretion. Interestingly, blockage of NF-κB pathway completely inhibited IL-8 and TNF-α secretion induced by all the pathotypes (Figures 8A,B). On the other hand, the blockage of ERK1/2 pathway did not cause complete inhibition of IL-8 and TNF-α secretion, but caused partial and differential inhibition. Thus, ERK1/2 pathway blockage caused the highest inhibition of IL-8 secretion induced by EAEC infection at 2 h (73%) and 4 h (63%) of infection, while the lowest inhibition was induced in cells infected for 2 h by *S. flexneri* (49%), EHEC (48.5%), or EIEC (47%), and at 4 h IL-8 secretion was inhibited 74–64% during infection by ETEC>EHEC>EPEC>EIEC>EAEC, while lowest inhibition (43%) was during the infection by *S. flexneri*. Remarkably, the ERK1/2 pathway inhibition appears to be compensated by the NF-κB pathway for IL-8 secretion because the remaining cytokine secretion during ERK1/2 blockage was completely inhibited by using both inhibitors (BAY 11-7082 and PD98059). Regarding the TNF-α secretion, blockage of ERK1/2 pathway completely inhibited TNF-α secretion induced by all the extracellular bacteria (EPEC, EHEC, ETEC, and EAEC) at 2 h of infection, but not those induced by the intracellular bacteria, whose inhibition were of 39 and 31% for EIEC and *S. flexneri*, respectively. Interestingly, inhibition of TNF-α secretion induced at 4 h by the different pathotypes due to the blockage of ERK1/2 pathway was less efficient mainly during infection by *S. flexneri* (32%), ETEC (33%), EIEC, and EAEC (43%) but efficiently inhibited during the infection by EHEC (61%) and EPEC (100%). Again, the remaining values of TNF-α secretion were completely inhibited when the inhibitors of both pathways were used.

## Discussion

We have developed an infection model of epithelial cells, which allowed reproduce the phenotypic features reported for the different diarrheagenic pathotypes using the same infection conditions such as multiplicity of infection and infection times. In these cells displaying the phenotypic feature induced by each phenotype were analyzed the nuclear translocation, kinetics of activation and modulation of NF-κB and ERK1/2 signaling pathways induced by different *E. coli* pathotypes. This model also allowed determine the outcome of the activation of these nuclear factors by quantifying the secretion of epithelial cytokines as well as the role of each signaling pathway by blocking the responses using inhibitors for NF-κB and/or ERK1/2. Thus, for the first time this work shows a comparison of inflammatory response of each pathotype on a specific cell line under the same condition and ensuring reproducibility of phenotype feature induced by each pathotype.

HT-29 cells allow reproducing the well-defined adherence pattern for EAEC, EPEC, and EHEC pathotypes, actin cytoskeletal rearrangement induced by EPEC and EHEC (pedestal formation), EAEC (cytoskeletal contraction and rounding cells), and EIEC/*Shigella* (intracellular actin tails); as well as adhesion of ETEC on epithelial cells without apparent cell damage (Kaper et al., 2004). HT-29 cells have been previously used in individual way in studies of the inflammatory response induced by some pathotypes (Sharma et al., 2006; Salazar-Gonzalez and Navarro-Garcia, 2011; Xue et al., 2014; Ye et al., 2015). HT-29 cells have several advantages for evaluating the activation of the inflammatory response regarding to other cell lines from intestinal linage such as Caco-2 and T84 cells. HT-29 cells have been proposed as an ideal model for evaluating the inflammatory response induced by EPEC than the T84 cells; produce a robust response of IL-8 secretion (Sharma et al., 2006). Indeed, here we have shown that HT-29 cell secreted equivalent levels of IL-8 that professional antigen presenting cells as the macrophages. Additionally, HT-29 cells express Gb-3, a Shiga toxin receptor, and are susceptible to Stx, while T84 cells do not expresses Gb-3 and thereby are resistant to the Stx effects such as inhibition of protein synthesis and ribotoxic stress response. This latter effect is relevant since there is upregulation of pro-inflammatory genes depending on Stx in Gb3-positive epithelial lines through the ribotoxic stress response (Thorpe et al., 1999; Smith et al., 2003). Furthermore, it has been demonstrated that TNF-α-stimulated HT-29 cells induce a strong NF-κB activation and subsequent IκB degradation, which did not occur in Caco-2 cells (Elewaut et al., 1999). Thus, HT-29 cells represent an appropriated model for our aim as well as to reproduce the specific pathogenesis schemes for each pathotype.

EPEC and mainly EHEC induced both p65 and ERK1/2 phosphorylation at early times (30 min) of infection. This correlates with previous studies, in which PAMPs as flagellin from EPEC (fliC), EHEC (H7), or the hemorrhagic coli pilus (HCP), promotes the early inflammatory response (Zhou et al., 2003; Miyamoto et al., 2006; Ledesma et al., 2010). However, at late times both p65 and ERK1/2 phosphorylation decreased in a time-dependent manner until it disappears. It has been described that after 60 min of infection, EPEC injects through T3SS non-LEE-effectors (Nle; Mills et al., 2013), related to NF-κB signaling pathway subversion, which could explain the decreased p65 phosphorylation in our infection model. Interestingly, p65 and ERK1/2 phosphorylation decrements were related to the inhibitory ability of these pathotypes, since co-stimulation experiments showed that EPEC/EHEC prevents TNF-α-induced p65 phosphorylation and EGF-induced ERK1/2 phosphorylation post-infection. At 4 h of infection, EHEC partially inhibited TNF-α-induced p65 phosphorylation and EGF-induced ERK1/2 phosphorylation, while a complete inhibition of both pathways was observed by EPEC infection. These inhibitory effects constitute a mechanism to subvert the host immune response for A/E pathotypes, in which it has been demonstrated that Nle-effectors cooperatively participate in suppress NF-κB activation; inhibiting IKKβ phosphorylation (NleE and NleB), (Nadler et al., 2010; Vossenkämper et al., 2010) or by direct enzymatic digestion of p65 subunit (NleC; Baruch et al., 2011; Pearson et al., 2011). Even though EPEC and EHEC have been involved in MAPK response inhibition, no effectors involved in specific inhibition of ERK1/2 have been described in EPEC or EHEC. Indeed, EPEC induces the cleavage of both p38 and JNK in NleD-dependent manner, but not that of ERK (Baruch et al., 2011). However, many studies describe a close association between ERK1/2 activity and an increased nuclear translocation of NF-κB. For instance, ERK regulates NF-κB activation through increasing IκB phosphorylation (Dhawan and Richmond, 2002) or NF-κB DNA binding activity (Briant et al., 1998). These inhibitory effects could explain the absence of signal translocation of NF-κB and ERK1/2 detected by confocal microscopy in response to EHEC and mainly EPEC infection at 4 h. However, although these pathotypes share many of these effectors, in our model, EHEC infection induced a slightly nuclear translocation of NF-κB but not EPEC. This difference might be given by Shiga toxin (Stx), a hallmark of EHEC, since it has been reported that Stx activates NF-κB in THP-1 cells (Sakiri et al., 1998). Thus, EPEC is more efficient than EHEC in subverting the inflammatory response, even when the EHEC genome encodes more than 40 effector proteins (Tobe et al., 2006), and EPEC encodes at least 21 (Iguchi et al., 2009). These data also might explain why EPEC infection of humans is not associated with strong inflammatory responses that compromises integrity of the epithelial barrier in spite of the strong attaching and effacing modifications.

While EPEC/EHEC pathotypes employ T3SS-dependent infection strategies, both ETEC and EAEC pathotypes employ different strategies, mainly based on their colonization factors followed by secretion of toxins that subsequently enter the host cell. It has been shown that ETEC infection activates both NF-κB and MAPK signaling pathways through mechanisms LT-dependent in HCT-8 (Wang et al., 2012) and T84 (Chutkan and Kuehn, 2011) IECs. In our study we also found that ETEC infection induced p65 phosphorylation from early times and it is maintained throughout the kinetics of infection. However, in co-stimulation experiments, ETEC infection appears to secrete potent anti-inflammatory factor(s), since it inhibited TNF-α-induced p65 phosphorylation. This inhibitory effect was seen from 30 min of infection, suggesting that a factor at early times is responsible for this inhibition. In this sense, the ST toxin does not need more than 30 min to interact with epithelial cells, because ST does not need to undergo intracellular traffic; its receptor is also the catalytic target, the guanylate cyclase (Schulz et al., 1990). Therefore, this model may be useful to study the role of ST in the inflammatory response. Contrastingly, EAEC infection induced strong p65 phosphorylation at early times comparable to TNF-α-induced NF-κB activation. This phenotype has been described in T84 and INT-407 IECs, in which EAEC infection activates both NF-κB and MAPK signaling pathways resulting in IL-8 production (Harrington et al., 2005; Khan et al., 2010), considering this pro-inflammatory response as an essential role in the EAEC-induced pathology. However, in our infection model, at late times some factor(s) from EAEC decreased p65 phosphorylation and this inhibition was kept in TNF-α-induced p65-phosphorylation. These data suggest the action of unknown anti-inflammatory factor(s), which probably help to counteract the exacerbated inflammatory response induced by this pathotype.

Interestingly and contrary to EPEC/EHEC, both ETEC and EAEC induced ERK1/2 phosphorylation only at late times (2 and 4 h respectively). These data are similar to those reported in epithelial cells infected with ETEC (HCT-8 cells) and EAEC (INT-407 cells; Khan et al., 2010; Wang et al., 2012). But none of these works mentioned the early inhibition induced by ETEC and EAEC on ERK1/2 phosphorylation at 30 min and 1 h post-infection. Furthermore, ETEC and mainly EAEC inhibited EGF-induced ERK1/2 phosphorylation mainly at early times. Therefore, this model is useful to identify potential news proteins or mechanisms related inhibition of ERK1/2 signaling pathway.

In contrast to extracellular bacteria, intracellular strains EIEC and mainly *S. flexneri* induced a strong p65 phosphorylation beyond the levels of TNF-α-induced p65 phosphorylation; correlating with the stronger p65 nuclear translocation induced by both intracellular strains in confocal microscopy experiments. Interestingly, co-stimulation with TNF-α further increased p65 phosphorylation in a differential way for these two bacteria: EIEC caused a partial synergistic effect, while *S. flexneri* caused a clear synergistic effect. It has been shown that LPS and IpgB2-OspB effectors from *S. flexneri* activate NF-κB pathway through recognition by cytosolic NOD1/CARD4 and NOD1/GEF-H1, respectively (Girardin et al., 2001; Fukazawa et al., 2008). Together in our infection model, these data suggest that both intracellular bacteria promote the NF-κB activation by cytosolic NLRs independently of the TNF-α-induced p65 phosphorylation. Besides these intracellular bacteria have no flagellum (Hale, 1991), therefore NF-κB activation is independent of TLR5-flagellin recognition. On the other hand, *S. flexneri* and mainly EIEC induced ERK1/2 phosphorylation at early and late times, but at intermediate times ERK1/2 phosphorylation drastically decreased. Co-stimulation experiments showed partial inhibitory effects in which, EGF-induced ERK1/2 phosphorylation does not reach 100% but at intermediate times ERK1/2 phosphorylation was not significantly different of those of late times of infection. It has been shown that the T3SS effector OspF from *S. flexneri* has a phosphothreonine lyase activity that irreversibly dephosphorylated MAPK (ERK1/2, p38, and JNK; Li et al., 2007) and OspF is also present in EIEC (Ud-Din and Wahid, 2014). Interestingly, OspF-induced MAPK dephosphorylation potentiates the activation of the NF-κB during *S. flexneri* infection (Reiterer et al., 2011). Although these intracellular strains are phylogenetically closely related, here we showed that *S. flexneri* is more efficient than EIEC inducing strong NF-κB activation and suppressing EGF-induced ERK1/2 phosphorylation.

In this model infection EHEC and mainly EPEC induced the lowest levels of IL-8 and TNF-α secretion compared to the other pathotypes, which correlated with the inhibitory effects on NF-κB and ERK1/2 activation. Interestingly, EPEC infection does not induced TNF-α secretion, which could result in a decreased inflammatory response (Bradley, 2008) compared to EHEC. Indeed, EHEC produce a hemorrhagic coli pilus (HCP), which is a potent inducer of IL-8 and TNF-α release through NF-κB and MAPK activation (Ledesma et al., 2010). Contrastingly, ETEC and mainly EAEC induced a strong IL-8 and TNF-α secretion, even when partial inhibitory effects of signaling pathways were observed. This difference in IL-8 secretion has been shown in isolates from patients with travelers' diarrhea, in which EAEC causes a greater response of IL-8 than ETEC (Huang et al., 2004). EAEC infection also induced the highest levels of TNF-α secretion. Remarkably, some stimuli, such as IL-1 or TNF, up-regulate IL-8 by more than 100-fold in human astrocytoma and alveolar epithelial cell lines (Kasahara et al., 1991; Brasier et al., 1998), suggesting a synergistic effect of EAEC by inducing high TNF-α and IL-8 secretion. Interestingly, intracellular strains induced a strong IL-8 and TNF-α secretion from early times of infection compared with the other pathotypes (except EAEC infection at 4 h), which correlates to the strong NF-κB activation and the intestinal pathology caused by these pathotypes, in spite of the inhibitory effects observed in ERK1/2 phosphorylation. In fact, significantly raised stool TNF-α has been detected in patients coursing shigellosis (de Silva et al., 1993). It is important to note that non-pathogenic strain HB101 did not induce nuclear translocation of NF-κB and ERK1/2 or modulation of signaling pathways in our infection model. This indicates that specific pathogenesis scheme of each pathotype are responsible for inducing the specific inflammatory responses observed in this model.

Notably, pre-treatment with a specific inhibitor of NF-κB (BAY 11-7082) completely prevents the IL-8 and TNF-α secretion induced by the different *E. coli* pathotypes. While the use of a specific inhibitor of ERK1/2 (PD98059) partially reduces the IL-8 secretion, however, it does not efficiently prevent TNF-α secretion induced by ETEC, EAEC, and mainly intracellular strains. This suggests that for all *E. coli* pathotypes, NF-κB signaling pathway is essential for the cytokine secretion, and partially dependent on ERK1/2 signaling pathway for the full induction of proinflammatory cytokine. It has been demonstrated, that the PD98059 inhibitor attenuates IkB degradation and the IL-8 expression, demonstrating that NF-κB activation is dependent on ERK1/2 (Savkovic et al., 2001). Additionally, it has been demonstrated that ERK1/2 inhibitor (PD98059) had no effect on LPS-induced TNF-α secretion in rat vascular smooth muscle cells. However, the p38 inhibitor (SB203580) partially blocks TNF-α secretion but a complete inhibition was observed with NF-κB inhibitors (PDTC and MG132; Yamakawa et al., 1999). All these suggest that in our model p38 MAPK could regulate TNF-α secretion in response to *E. coli* pathotypes infection.

**Figure 9:**
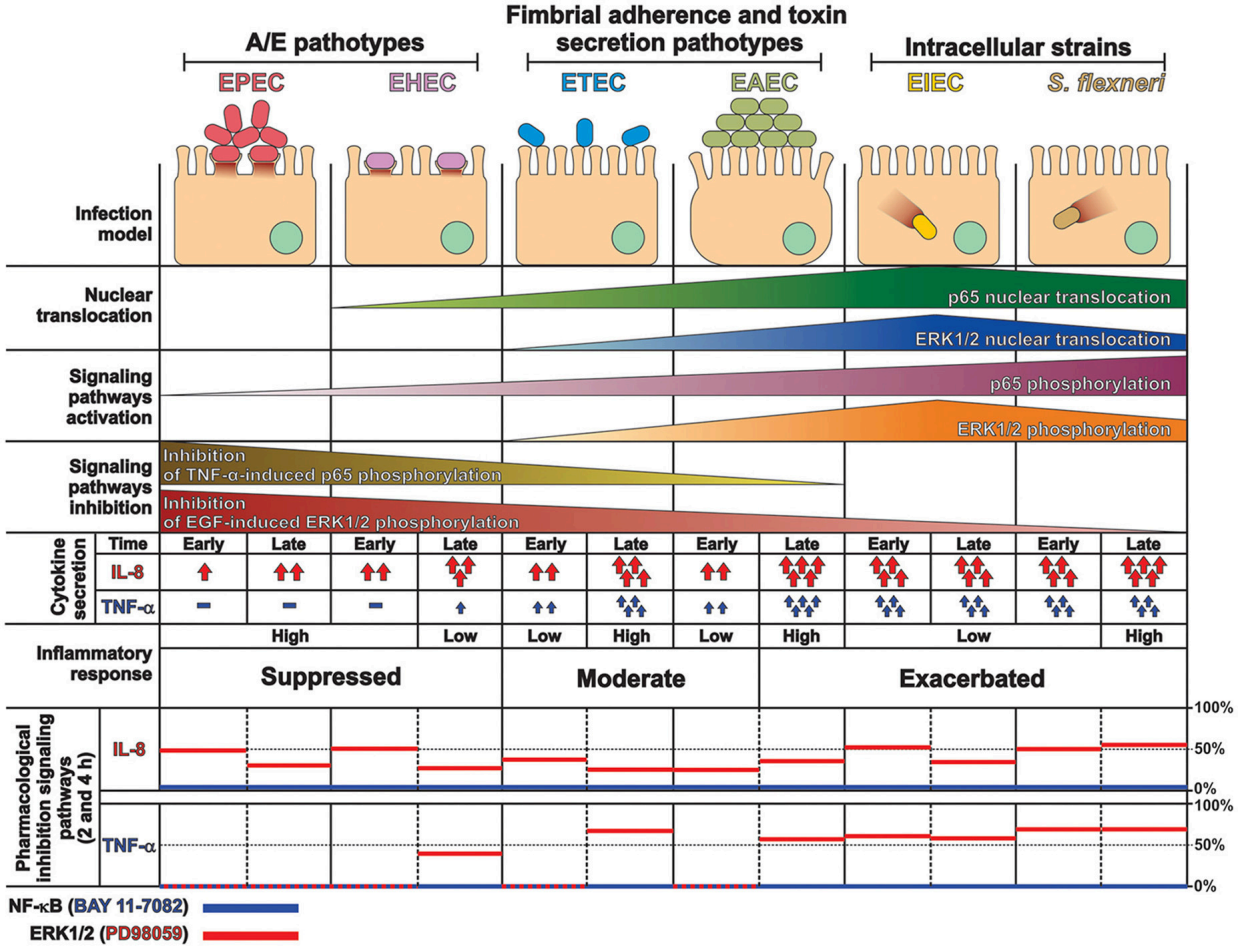
**Summary of the inflammatory responses induced by the different pathotype on epithelial cells**. HT-29 cells differentially responded to the infection by the different pathotypes. In spite of their differences, the pathotype can also be divided in three groups according to their inflammatory response: (i) EPEC and EHEC that inject effectors to cause A/E lesion, which are able to inhibit NF-κB and ERK1/2 pathways, and cytokine secretion; (ii) EAEC and ETEC with fimbrial adherence and toxin secretion with a moderate inhibition of both pathways but high cytokines secretion through autocrine cytokine regulation; and (iii) *S. flexneri* and EIEC, the intracellular bacteria, that induce the highest pathways activation and cytokines secretion by using different activation mechanisms. The secretion of IL-8 and TNF-α induced by the different *E. coli* pathotypes was partially inhibited by ERK1/2 inhibitor (PD98059) and completely inhibited by NF-κB inhibitor (BAY 11-7082).

These data together, provides a comprehensive and detailed analysis that allowed us to compare how the different pathogenesis schemes of *E. coli* pathotypes manipulate signaling pathways related to the inflammatory response, which leads to a specific proinflammatory cytokine secretion for each pathotype in a cell model infection (see Figure 9) that reproduce the hallmarks of infection of each pathotype. Our findings let us to propose that two events can be playing a role in these differential responses: an unbalanced response between NF-κB and ERK1/2 pathways and differential secretion of TNF-α, which could synergize the inflammatory response including IL-8 secretion (Figure 9).

## Author contributions

JS performed experiments and helped to write the paper. GT helped to perform some experiments. FN wrote the paper and provided financial support.

### Conflict of interest statement

The authors declare that the research was conducted in the absence of any commercial or financial relationships that could be construed as a potential conflict of interest.

## References

[B1] Arthur J. S. Ley S. C. (2013). Mitogen-activated protein kinases in innate immunity. Nat. Rev. Immunol. 13, 679–692. 10.1038/nri3495 23954936

[B2] Baruch K. Gur-Arie L. Nadler C. Koby S. Yerushalmi G. Ben-Neriah Y.. (2011). Metalloprotease type III effectors that specifically cleave JNK and NF-κB. EMBO J. 30, 221–231. 10.1038/emboj.2010.297 21113130 PMC3020117

[B3] Boyer H. W. Roulland-Dussoix D. (1969). A complementation analysis of the restriction and modification of DNA in *Escherichia coli*. J. Mol. Biol. 41, 459–472. 10.1016/0022-2836(69)90288-5 4896022

[B4] Bradley J. R. (2008). TNF-mediated inflammatory disease. J. Pathol. 214, 149–160. 10.1002/path.2287 18161752

[B5] Brasier A. R. Jamaluddin M. Casola A. Duan W. Shen Q. Garofalo R. P. (1998). A promoter recruitment mechanism for tumor necrosis factor-alpha-induced interleukin-8 transcription in type II pulmonary epithelial cells. Dependence on nuclear abundance of Rel A, NF-κB1, and c-Rel transcription factors. J. Biol. Chem. 273, 3551–3561. 9452482 10.1074/jbc.273.6.3551

[B6] Briant L. Robert-Hebmann V. Sivan V. Brunet A. Pouyssegur J. Devaux C. (1998). Involvement of extracellular signal-regulated kinase module in HIV-mediated CD4 signals controlling activation of nuclear factor-κ B and AP-1 transcription factors. J. Immunol. 160, 1875–1885. 9469449

[B7] Chutkan H. Kuehn M. J. (2011). Context-dependent activation kinetics elicited by soluble versus outer membrane vesicle-associated heat-labile enterotoxin. Infect. Immun. 79, 3760–3769. 10.1128/IAI.05336-11 21708992 PMC3165487

[B8] Croxen M. A. Law R. J. Scholz R. Keeney K. M. Wlodarska M. Finlay B. B. (2013). Recent advances in understanding enteric pathogenic *Escherichia coli*. Clin. Microbiol. Rev. 26, 822–880. 10.1128/CMR.00022-13 24092857 PMC3811233

[B9] de Silva D. G. Mendis L. N. Sheron N. Alexander G. J. Candy D. C. Chart H.. (1993). Concentrations of interleukin 6 and tumour necrosis factor in serum and stools of children with *Shigella dysenteriae* 1 infection. Gut 34, 194–198. 10.1136/gut.34.2.194 8432472 PMC1373969

[B10] Dhawan P. Richmond A. (2002). A novel NF-κ B-inducing kinase-MAPK signaling pathway up-regulates NF-κ B activity in melanoma cells. J. Biol. Chem. 277, 7920–7928. 10.1074/jbc.M112210200 11773061 PMC2668260

[B11] DuPont H. L. Formal S. B. Hornick R. B. Snyder M. J. Libonati J. P. Sheahan D. G.. (1971). Pathogenesis of *Escherichia coli* diarrhea. N. Engl. J. Med. 285, 1–9. 10.1056/NEJM197107012850101 4996788

[B12] DuPont H. L. Levine M. M. Hornick R. B. Formal S. B. (1989). Inoculum size in shigellosis and implications for expected mode of transmission. J. Infect. Dis. 159, 1126–1128. 10.1093/infdis/159.6.1126 2656880

[B13] Elewaut D. DiDonato J. A. Kim J. M. Truong F. Eckmann L. Kagnoff M. F. (1999). NF-κ B is a central regulator of the intestinal epithelial cell innate immune response induced by infection with enteroinvasive bacteria. J. Immunol. 163, 1457–1466. 10415047

[B14] Estrada-Garcia T. Navarro-Garcia F. (2012). Enteroaggregative *Escherichia coli* pathotype: a genetically heterogeneous emerging foodborne enteropathogen. FEMS Immunol. Med. Microbiol. 66, 281–298. 10.1111/j.1574-695X.2012.01008.x 22775224

[B15] Evans D. G. Silver R. P. Evans D. J. Jr. Chase D. G. Gorbach S. L. (1975). Plasmid-controlled colonization factor associated with virulence in *Escherichia coli* enterotoxigenic for humans. Infect. Immun. 12, 656–667. 1100526 10.1128/iai.12.3.656-667.1975 PMC415337

[B16] Fukazawa A. Alonso C. Kurachi K. Gupta S. Lesser C. F. McCormick B. A.. (2008). GEF-H1 mediated control of NOD1 dependent NF-κB activation by *Shigella* effectors. PLoS Pathog. 4:e1000228. 10.1371/journal.ppat.1000228 19043560 PMC2583055

[B17] Gasparini C. Feldmann M. (2012). NF-κB as a target for modulating inflammatory responses. Curr. Pharm. Des. 18, 5735–5745. 10.2174/138161212803530763 22726116

[B18] Girardin S. E. Tournebize R. Mavris M. Page A. L. Li X. Stark G. R.. (2001). CARD4/Nod1 mediates NF-κB and JNK activation by invasive *Shigella flexneri*. EMBO Rep. 2, 736–742. 10.1093/embo-reports/kve155 11463746 PMC1083992

[B19] Hale T. L. (1991). Genetic basis of virulence in *Shigella* species. Microbiol. Rev. 55, 206–224. 1886518 10.1128/mr.55.2.206-224.1991 PMC372811

[B20] Harrington S. M. Strauman M. C. Abe C. M. Nataro J. P. (2005). Aggregative adherence fimbriae contribute to the inflammatory response of epithelial cells infected with enteroaggregative *Escherichia coli*. Cell. Microbiol. 7, 1565–1578. 10.1111/j.1462-5822.2005.00588.x 16207244

[B21] Huang D. B. DuPont H. L. Jiang Z. D. Carlin L. Okhuysen P. C. (2004). Interleukin-8 response in an intestinal HCT-8 cell line infected with enteroaggregative and enterotoxigenic *Escherichia coli*. Clin. Diagn. Lab. Immunol. 11, 548–551. 10.1128/cdli.11.3.548-551.2004 15138180 PMC404585

[B22] Iguchi A. Thomson N. R. Ogura Y. Saunders D. Ooka T. Henderson I. R.. (2009). Complete genome sequence and comparative genome analysis of enteropathogenic *Escherichia coli* O127:H6 strain E2348/69. J. Bacteriol. 191, 347–354. 10.1128/JB.01238-08 18952797 PMC2612414

[B23] Jijon H. B. Buret A. Hirota C. L. Hollenberg M. D. Beck P. L. (2012). The EGF receptor and HER2 participate in TNF-α-dependent MAPK activation and IL-8 secretion in intestinal epithelial cells. Mediators Inflamm. 2012:207398. 10.1155/2012/207398 22988345 PMC3440955

[B24] Kagnoff M. F. Eckmann L. (1997). Epithelial cells as sensors for microbial infection. J. Clin. Invest. 100, 6–10. 10.1172/JCI119522 9202050 PMC508158

[B25] Kaper J. B. Nataro J. P. Mobley H. L. (2004). Pathogenic *Escherichia coli*. Nat. Rev. Microbiol. 2, 123–140. 10.1038/nrmicro818 15040260

[B26] Kasahara T. Mukaida N. Yamashita K. Yagisawa H. Akahoshi T. Matsushima K. (1991). IL-1 and TNF-α induction of IL-8 and monocyte chemotactic and activating factor (MCAF) mRNA expression in a human astrocytoma cell line. Immunology 74, 60–67. 1937574 PMC1384672

[B27] Kawai T. Akira S. (2010). The role of pattern-recognition receptors in innate immunity: update on Toll-like receptors. Nat. Immunol. 11, 373–384. 10.1038/ni.1863 20404851

[B28] Khan K. Konar M. Goyal A. Ghosh S. (2010). Enteroaggregative *Escherichia coli* infection induces IL-8 production via activation of mitogen-activated protein kinases and the transcription factors NF-κB and AP-1 in INT-407 cells. Mol. Cell. Biochem. 337, 17–24. 10.1007/s11010-009-0282-3 19898747

[B29] Labrec E. H. Schneider H. Magnani T. J. Formal S. B. (1964). Epithelial cell penetration as an essential step in the pathogenesis of bacillary dysentery. J. Bacteriol. 88, 1503–1518. 16562000 10.1128/jb.88.5.1503-1518.1964 PMC277436

[B30] Lanata C. F. Fischer-Walker C. L. Olascoaga A. C. Torres C. X. Aryee M. J. Black R. E.. (2013). Global causes of diarrheal disease mortality in children < 5 years of age: a systematic review. PLoS ONE 8:e72788. 10.1371/journal.pone.0072788 24023773 PMC3762858

[B31] Ledesma M. A. Ochoa S. A. Cruz A. Rocha-Ramirez L. M. Mas-Oliva J. Eslava C. A.. (2010). The hemorrhagic coli pilus (HCP) of *Escherichia coli* O157:H7 is an inducer of proinflammatory cytokine secretion in intestinal epithelial cells. PLoS ONE 5:e12127. 10.1371/journal.pone.0012127 20711431 PMC2920817

[B32] Levine M. M. Bergquist E. J. Nalin D. R. Waterman D. H. Hornick R. B. Young C. R.. (1978). *Escherichia coli* strains that cause diarrhoea but do not produce heat-labile or heat-stable enterotoxins and are non-invasive. Lancet 1, 1119–1122. 10.1016/S0140-6736(78)90299-4 77415

[B33] Li H. Xu H. Zhou Y. Zhang J. Long C. Li S.. (2007). The phosphothreonine lyase activity of a bacterial type III effector family. Science 315, 1000–1003. 10.1126/science.1138960 17303758

[B34] Liu L. Johnson H. L. Cousens S. Perin J. Scott S. Lawn J. E.. (2012). Global, regional, and national causes of child mortality: an updated systematic analysis for 2010 with time trends since 2000. Lancet 379, 2151–2161. 10.1016/S0140-6736(12)60560-1 22579125

[B35] McDaniel T. K. Jarvis K. G. Donnenberg M. S. Kaper J. B. (1995). A genetic locus of enterocyte effacement conserved among diverse enterobacterial pathogens. Proc. Natl. Acad. Sci. U.S.A. 92, 1664–1668. 10.1073/pnas.92.5.1664 7878036 PMC42580

[B36] Mills E. Baruch K. Aviv G. Nitzan M. Rosenshine I. (2013). Dynamics of the type III secretion system activity of enteropathogenic *Escherichia coli*. MBio 4:e00303–13. 10.1128/mBio.00303-13 23900171 PMC3735188

[B37] Miyamoto Y. Iimura M. Kaper J. B. Torres A. G. Kagnoff M. F. (2006). Role of Shiga toxin versus H7 flagellin in enterohaemorrhagic *Escherichia coli* signalling of human colon epithelium *in vivo*. Cell. Microbiol. 8, 869–879. 10.1111/j.1462-5822.2005.00673.x 16611235

[B38] Moreno A. C. Ferreira L. G. Martinez M. B. (2009). Enteroinvasive *Escherichia coli* vs. *Shigella flexneri*: how different patterns of gene expression affect virulence. FEMS Microbiol. Lett. 301, 156–163. 10.1111/j.1574-6968.2009.01815.x 19889166

[B39] Nadler C. Baruch K. Kobi S. Mills E. Haviv G. Farago M.. (2010). The type III secretion effector NleE inhibits NF-κB activation. PLoS Pathog. 6:e1000743. 10.1371/journal.ppat.1000743 20126447 PMC2813277

[B40] Nataro J. P. Deng Y. Cookson S. Cravioto A. Savarino S. J. Guers L. D.. (1995). Heterogeneity of enteroaggregative *Escherichia coli* virulence demonstrated in volunteers. J. Infect. Dis. 171, 465–468. 10.1093/infdis/171.2.465 7844392

[B41] Pearson J. S. Riedmaier P. Marches O. Frankel G. Hartland E. L. (2011). A type III effector protease NleC from enteropathogenic *Escherichia coli* targets NF-κB for degradation. Mol. Microbiol. 80, 219–230. 10.1111/j.1365-2958.2011.07568.x 21306441 PMC3178796

[B42] Reiterer V. Grossniklaus L. Tschon T. Kasper C. A. Sorg I. Arrieumerlou C. (2011). *Shigella flexneri* type III secreted effector OspF reveals new crosstalks of proinflammatory signaling pathways during bacterial infection. Cell. Signal. 23, 1188–1196. 10.1016/j.cellsig.2011.03.006 21402152

[B43] Riley L. W. Remis R. S. Helgerson S. D. McGee H. B. Wells J. G. Davis B. R.. (1983). Hemorrhagic colitis associated with a rare *Escherichia coli* serotype. N. Engl. J. Med. 308, 681–685. 10.1056/NEJM198303243081203 6338386

[B44] Sakiri R. Ramegowda B. Tesh V. L. (1998). Shiga toxin type 1 activates tumor necrosis factor-alpha gene transcription and nuclear translocation of the transcriptional activators nuclear factor-κB and activator protein-1. Blood 92, 558–566. 9657756

[B45] Salazar-Gonzalez H. Navarro-Garcia F. (2011). Intimate adherence by enteropathogenic *Escherichia coli* modulates TLR5 localization and proinflammatory host response in intestinal epithelial cells. Scand. J. Immunol. 73, 268–283. 10.1111/j.1365-3083.2011.02507.x 21204905

[B46] Sanchez-Villamil J. Navarro-Garcia F. (2015). Role of virulence factors on host inflammatory response induced by diarrheagenic *Escherichia coli* pathotypes. Future Microbiol. 10, 1009–1033. 10.2217/fmb.15.17 26059623

[B47] Savkovic S. D. Ramaswamy A. Koutsouris A. Hecht G. (2001). EPEC-activated ERK1/2 participate in inflammatory response but not tight junction barrier disruption. Am. J. Physiol. Gastrointest. Liver Physiol. 281, G890–898. 11557508 10.1152/ajpgi.2001.281.4.G890

[B48] Schulz S. Green C. K. Yuen P. S. Garbers D. L. (1990). Guanylyl cyclase is a heat-stable enterotoxin receptor. Cell 63, 941–948. 10.1016/0092-8674(90)90497-3 1701694

[B49] Sharma R. Tesfay S. Tomson F. L. Kanteti R. P. Viswanathan V. K. Hecht G. (2006). Balance of bacterial pro- and anti-inflammatory mediators dictates net effect of enteropathogenic *Escherichia coli* on intestinal epithelial cells. Am. J. Physiol. Gastrointest. Liver Physiol. 290, G685–G694. 10.1152/ajpgi.00404.2005 16322091

[B50] Smith W. E. Kane A. V. Campbell S. T. Acheson D. W. Cochran B. H. Thorpe C. M. (2003). Shiga toxin 1 triggers a ribotoxic stress response leading to p38 and JNK activation and induction of apoptosis in intestinal epithelial cells. Infect. Immun. 71, 1497–1504. 10.1128/IAI.71.3.1497-1504.2003 12595468 PMC148871

[B51] Thorpe C. M. Hurley B. P. Lincicome L. L. Jacewicz M. S. Keusch G. T. Acheson D. W. (1999). Shiga toxins stimulate secretion of interleukin-8 from intestinal epithelial cells. Infect. Immun. 67, 5985–5993. 10531258 10.1128/iai.67.11.5985-5993.1999 PMC96984

[B52] Tobe T. Beatson S. A. Taniguchi H. Abe H. Bailey C. M. Fivian A.. (2006). An extensive repertoire of type III secretion effectors in *Escherichia coli* O157 and the role of lambdoid phages in their dissemination. Proc. Natl. Acad. Sci. U.S.A. 103, 14941–14946. 10.1073/pnas.0604891103 16990433 PMC1595455

[B53] Ud-Din A. Wahid S. (2014). Relationship among *Shigella* spp. and enteroinvasive *Escherichia coli* (EIEC) and their differentiation. Braz. J. Microbiol. 45, 1131–1138. 10.1590/S1517-83822014000400002 25763015 PMC4323284

[B54] Vossenkämper A. Marches O. Fairclough P. D. Warnes G. Stagg A. J. Lindsay J. O.. (2010). Inhibition of NF-κB signaling in human dendritic cells by the enteropathogenic *Escherichia coli* effector protein NleE. J. Immunol. 185, 4118–4127. 10.4049/jimmunol.1000500 20833837

[B55] Wang X. Gao X. Hardwidge P. R. (2012). Heat-labile enterotoxin-induced activation of NF-κB and MAPK pathways in intestinal epithelial cells impacts enterotoxigenic *Escherichia coli* (ETEC) adherence. Cell. Microbiol. 14, 1231–1241. 10.1111/j.1462-5822.2012.01793.x 22452361 PMC3391543

[B56] Xue Y. Zhang H. Wang H. Hu J. Du M. Zhu M. J. (2014). Host inflammatory response inhibits *Escherichia coli* O157:H7 adhesion to gut epithelium through augmentation of mucin expression. Infect. Immun. 82, 1921–1930. 10.1128/IAI.01589-13 24566630 PMC3993425

[B57] Yamakawa T. Eguchi S. Matsumoto T. Yamakawa Y. Numaguchi K. Miyata I.. (1999). Intracellular signaling in rat cultured vascular smooth muscle cells: roles of nuclear factor-κB and p38 mitogen-activated protein kinase on tumor necrosis factor-alpha production. Endocrinology 140, 3562–3572. 10.1210/en.140.8.3562 10433212

[B58] Ye J. Pan Q. Shang Y. Wei X. Peng Z. Chen W.. (2015). Core 2 mucin-type O-glycan inhibits EPEC or EHEC O157:H7 invasion into HT-29 epithelial cells. Gut Pathog. 7:31. 10.1186/s13099-015-0078-9 26677400 PMC4681020

[B59] Zhou X. Girón J. A. Torres A. G. Crawford J. A. Negrete E. Vogel S. N.. (2003). Flagellin of enteropathogenic *Escherichia coli* stimulates interleukin-8 production in T84 cells. Infect. Immun. 71, 2120–2129. 10.1128/IAI.71.4.2120-2129.2003 12654834 PMC152053

